# Regulation of colonic epithelial cell homeostasis by mTORC1

**DOI:** 10.1038/s41598-020-70655-1

**Published:** 2020-08-14

**Authors:** Takenori Kotani, Jajar Setiawan, Tasuku Konno, Noriko Ihara, Saki Okamoto, Yasuyuki Saito, Yoji Murata, Tetsuo Noda, Takashi Matozaki

**Affiliations:** 1 Division of Molecular and Cellular Signaling, Department of Biochemistry and Molecular Biology, Kobe University Graduate School of Medicine, Kobe, Japan; 2 Department of Physiology, Faculty of Medicine, Public Health, and Nursing, Universitas Gadjah Mada, Yogyakarta, Indonesia; 3 Department of Cell Biology, Cancer Institute, Japanese Foundation for Cancer Research, Tokyo, Japan

**Keywords:** Gastrointestinal hormones, TOR signalling, Intestinal stem cells

## Abstract

Cell signaling important for homeostatic regulation of colonic epithelial cells (CECs) remains poorly understood. Mammalian target of rapamycin complex 1 (mTORC1), a protein complex that contains the serine-threonine kinase mTOR, mediates signaling that underlies the control of cellular functions such as proliferation and autophagy by various external stimuli. We here show that ablation of tuberous sclerosis complex 2 (Tsc2), a negative regulator of mTORC1, specifically in intestinal epithelial cells of mice resulted in increased activity of mTORC1 of, as well as increased proliferative activity of, CECs. Such Tsc2 ablation also reduced the population of Lgr5-positive colonic stem cells and the expression of Wnt target genes in CECs. The stimulatory phosphorylation of the kinase Akt and inhibitory phosphorylation of glycogen synthase kinase 3β were both markedly decreased in the colon of the Tsc2 conditional knockout (CKO) mice. Development of colonic organoids with cryptlike structures was enhanced for Tsc2 CKO mice compared with control mice. Finally, Tsc2 CKO mice manifested increased susceptibility to dextran sulfate sodium–induced colitis. Our results thus suggest that mTORC1 activity promotes the proliferation of, as well as the expression of Wnt target genes in, CECs and thereby contributes to colonic organogenesis and homeostasis.

## Introduction

Both the small intestine and colon of mammals are important for food digestion and the absorption of nutrients, water, and electrolytes. The intestinal epithelium, in particular, plays a key role in these functions, although the structure and cell components of the epithelium differ between the small intestine and colon. For instance, the epithelium of the small intestine forms large villi that are not present in the colon, whereas crypts are much deeper in the colon than in the small intestine. In addition, the small intestinal epithelium contains Paneth cells, which produce antimicrobial peptides as well as Wnt ligands important for maintenance of intestinal stem cells (ISCs)^1,2^, whereas the colonic epithelium lacks Paneth cells. Instead, GLI1-expressing cells in the subepithelial mesenchyme have been thought to be the source of Wnt ligands for the maintenance of ISCs in the colon^3^. The population of mucin-producing goblet cells is also much larger in the colonic epithelium than in the small intestinal epithelium, presumably because of the larger number of microbes in the lumen of the colon^4^. Finally, inflammation as well as cancer develop preferentially in the epithelium of the colon compared with that of the small intestine, although the mechanisms underlying this difference remain largely unknown.

The turnover of intestinal epithelial cells (IECs) is relatively rapid—3 to 5 days for the small intestine and 5 to 7 days for the colon—and these cells are generated continuously throughout adulthood by ISCs, which are located in the stem cell niche at the bottom of intestinal crypts^5^. In the small intestine, ISCs generate proliferating progeny—known as transient amplifying (TA) cells—that migrate out of the stem cell niche, divide frequently, and terminally differentiate into distinct functional cell lineages of the epithelium including Paneth cells, goblet cells, and absorptive enterocytes^5^. These terminally differentiated IECs, except Paneth cells, migrate upwards along each intestinal villus and are eventually shed from the villus tip into the gut lumen^5^. Such homeostasis of the intestinal epithelium is thought to be coordinately regulated by various signaling molecules^2^. For example, we have shown that Src family kinases promote the proliferation and turnover of IECs through activation of the small GTPase Rac or the transcription co-activator YAP^6^. In addition, we and others have shown that the Ras–MAPK (mitogen–activated protein kinase) signaling pathway promotes the generation of both absorptive enterocytes and goblet cells^7, 8^, likely by counteracting the Wnt signaling pathway^9^. In addition to their role in the maintenance of ISCs, Wnt ligands are important for the generation of Paneth cells in the crypt^10^. The Notch signaling pathway is also thought to be important for maintenance of the ISC pool and for regulating the balance of secretory and absorptive cell lineage^11,12^.

In contrast to IECs of the small intestine, the molecular mechanisms underlying the rapid turnover and homeostasis of colonic epithelial cells (CECs) remain poorly understood. Mammalian target of rapamycin (mTOR) is a serine-threonine kinase that forms two distinct multiprotein complexes known as mTOR complex 1 (mTORC1) and mTORC2^13^. The activity of mTORC1 is controlled by various upstream signals such as growth factors, nutrients, and stress. For instance, growth factors such as insulin induce the activation of phosphatidylinositol 3-kinase and the downstream serine-threonine kinase Akt. Akt activates the small GTP-binding protein Rheb through phosphorylation and consequent inhibition of tuberous sclerosis complex (Tsc) 2, a GTPase-activating protein for Rheb, and Rheb then activates mTORC1. Such Rheb-mediated activation of mTORC1 results in the phosphorylation of ribosomal protein S6 kinase and the translational repressor protein 4E-BP1 and thereby regulates a variety of cellular processes such as protein synthesis, autophagy, and cell aging^13^. Whereas intestinal tumorigenesis driven by mutations in the *Apc* gene requires the activity of mTORC1^14,15^, the physiological role of mTORC1 in homeostatic regulation of CECs has remained unclear.

We recently showed that mTORC1 is important for the proliferation and migration of IECs in the small intestine^16^, whereas it has been poorly understood whether mTORC1 regulates turnover and homeostasis of CECs. Understanding the physiological role of mTORC1 in CECs may help development of new diagnostic methods or treatments for colonic inflammation and cancers. Here, with the use of IEC-specific Tsc2 conditional knockout (CKO) mice, we examined the potential role of mTORC1 in the homeostatic regulation of CECs.

## Results

### Activation of mTORC1 in CECs of IEC-specific Tsc2 knockout mice

**Figure 1:**
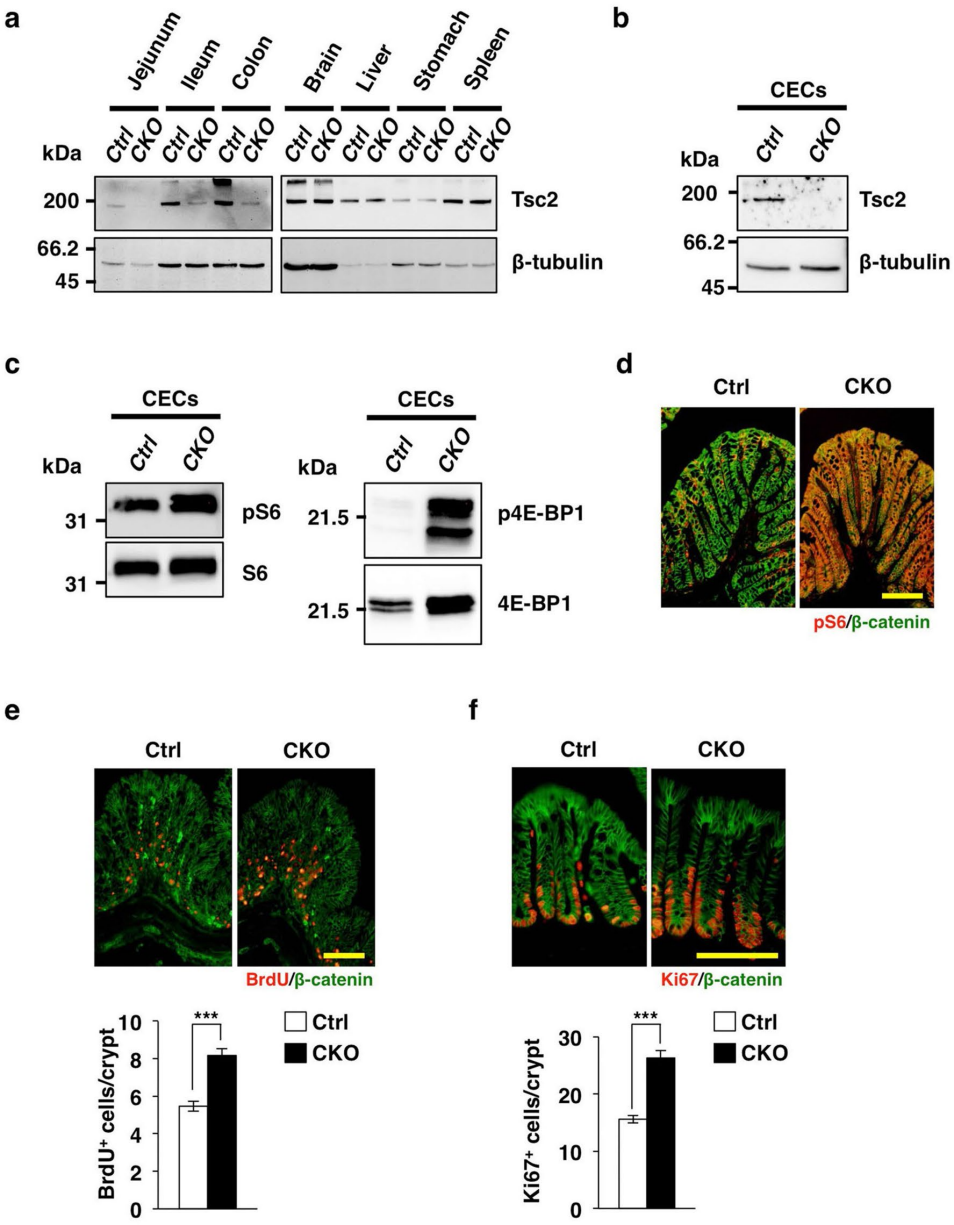
Increased proliferative activity of CECs in Tsc2 CKO mice. (**a**) Immunoblot analysis of lysates of the intestine and other organs from 9-week-old control (Ctrl) or Tsc2 CKO mice with antibodies to Tsc2 and to β-tubulin (loading control). (**b**) Immunoblot analysis of lysates of CECs from 14-week-old control or Tsc2 CKO mice with antibodies to Tsc2 and to β-tubulin. (**c**) Immunoblot analysis of lysates of CECs from 14-week-old control or Tsc2 CKO mice with antibodies to phosphorylated (p) or total forms of ribosomal protein S6 or 4E-BP1. (**d**) Immunohistofluorescence analysis of frozen sections of the colon from 10-week-old control or Tsc2 CKO mice with antibodies to pS6 (red) and to β-catenin (green). Scale bar, 100 μm. (**e**) Immunohistofluorescence analysis of frozen sections of the colon from control or Tsc2 CKO mice with antibodies to BrdU (red) and to β-catenin (green) at 2 h after BrdU injection. Representative images as well as quantitation of the number of BrdU-positive cells per crypt are shown. Scale bar, 100 μm. Quantitative data are means ± s.e. for 90 crypts from three control and three Tsc2 CKO mice at 14- to 16-week-old. ***P < 0.001 (Student’s *t* test). (**f**) Immunohistofluorescence analysis of frozen sections of the colon from control or Tsc2 CKO mice with antibodies to Ki67 (red) and to β-catenin (green). Representative images as well as quantitation of the number of Ki67-positive cells per crypt are shown. Scale bar, 100 μm. Quantitative data are means ± s.e. for 80 crypts from three control and three Tsc2 CKO mice at 10- to 14-week-old. ***P < 0.001 (Student’s *t* test). Uncropped immunoblot data of **(a)**, **(b)**, and **(c)** are shown in Supplementary Fig. S8.

The activity of mTORC1 is negatively regulated by Tsc1/2 in the basal state^13^. To evaluate the importance of mTORC1 in homeostatic regulation of CECs, we therefore generated Tsc2 CKO mice by crossing mice homozygous for a floxed *Tsc2* allele^16,17^ with those harboring a transgene for Cre recombinase under the control of the villin gene promoter. Immunoblot analysis showed that the abundance of Tsc2 protein in the jejunum, ileum, and colon of Tsc2 CKO mice was markedly reduced compared with that for control mice, whereas it was unaffected in other organs (Fig. 1a). Immunoblot analysis also failed to detect Tsc2 protein in CECs isolated from the colon of Tsc2 CKO mice (Fig. 1b), suggesting that Tsc2 was specifically ablated in CECs of the mutant mice. Moreover, immunoblot analysis showed that the amount and phosphorylation level of ribosomal protein S6 and 4E-BP1, which reflect mTORC1 activity^18,19^, were markedly increased in CECs from Tsc2 CKO mice (Fig. 1c). Immunohistofluorescence analysis also revealed that staining of phosphorylated S6 was mainly observed in the upper portion of the colonic crypt (where mature differentiated cells reside) in the control mice. In contrast, more prominent staining for phosphorylated S6 was observed throughout the colonic crypts of Tsc2 CKO mice compared with those of control mice (Fig. 1d). These results thus indicated that Tsc2 ablation resulted in hyperactivation of mTORC1 in CECs of Tsc2 CKO mice.

### Increased proliferative activity of CECs in Tsc2 CKO mice

Tsc2 CKO mice appeared healthy at birth, were born in the expected Mendelian ratio, and did not manifest any overt developmental defects (Supplementary Fig. S1a). Histologic examination of the colon revealed no marked difference in crypt architecture or epithelial cell morphology between control and Tsc2 CKO mice at 16 weeks of age (Supplementary Fig. S1b). Moreover, immunohistofluorescence analysis revealed that the number of Muc2-positive epithelial cells, a major component of the colonic epithelium, did not differ between Tsc2 CKO and control mice (Supplementary Fig. S1c). Given that mTORC1 plays an important role in the regulation of cell proliferation, we next examined the incorporation of bromodeoxyuridine (BrdU) into CECs of Tsc2 CKO mice. At 2 h after BrdU injection, the number of BrdU-positive CECs in the bottom portion of crypts in the colon was markedly increased for Tsc2 CKO mice compared with control mice (Fig. 1e). In addition, immunostaining for Ki67, a marker of cell proliferation^20^, showed a marked increase in the number of Ki67-positive CECs in colonic crypts of Tsc2 CKO mice (Fig. 1f). These results thus suggested that ablation of Tsc2 results in promotion of the proliferative activity of CECs, particularly in the bottom portion of the crypt, in vivo.

### Marked reduction in the number of leucine-rich repeat–containing G protein–coupled receptor 5 (Lgr5)–positive colonic stem cells in Tsc2 CKO mice

**Figure 2:**
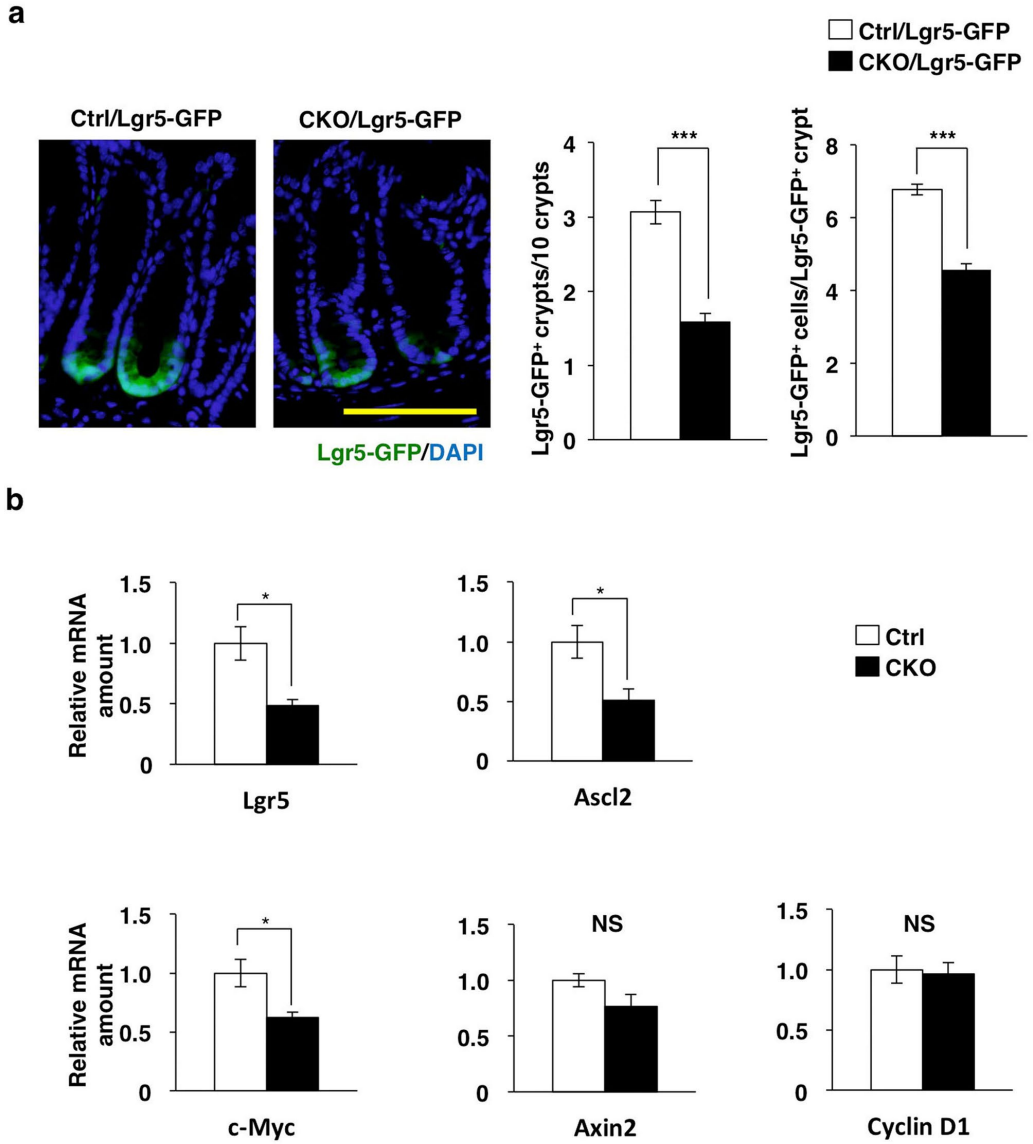
Marked reduction in the number of Lgr5-positive stem cells in the colon of Tsc2 CKO mice. (**a**) Frozen sections of the colon from control/Lgr5-GFP and Tsc2 CKO/Lgr5-GFP mice at 8- to 10-week-old were stained with 4′,6-diamidino-2-phenylindole (DAPI, blue) to detect nuclei and examined for GFP fluorescence (green). Representative images (scale bar, 100 μm) as well as quantitative analysis both of the number of Lgr5-GFP–positive crypts per 10 crypts (means ± s.e. for 994 crypts from three control/Lgr5-GFP mice and 1,002 crypts from three Tsc2 CKO/Lgr5-GFP mice) and of the number of Lgr5-GFP–positive cells per Lgr5-GFP–positive crypt (means ± s.e. for 90 Lgr5-GFP–positive crypts from three control/Lgr5-GFP and three Tsc2 CKO/Lgr5-GFP mice) are shown. ***P < 0.001 (Student’s *t* test). (**b**) Quantitative RT-PCR analysis of Lgr5, Ascl2, c-Myc, Axin2, and cyclin D1 mRNAs in CECs isolated from control and Tsc2 CKO mice at 9- to 12-week-old. The amount of each mRNA was normalized by that of Hprt1 mRNA and then expressed relative to the normalized value for control mice. Data are means ± s.e. from three separate experiments. *P < 0.05; NS, not significant (Student’s *t* test).

In the colonic epithelium, leucine-rich repeat–containing G protein–coupled receptor 5 (Lgr5)–positive stem cells at the base of each crypt are thought to undergo self-renewal and to generate TA cells as highly proliferative progeny^5,21^. Given the hyperproliferative activity of CECs in crypts of Tsc2 CKO mice, we next determined the number of colonic stem cells (CSCs) in these animals. We crossed *Lgr5-Gfp-CreERT2* (Lgr5-GFP) mice, in which some Lgr5-positive stem cells express green fluorescent protein (GFP)^21^, with either control or Tsc2 CKO mice in order to identify Lgr5-positive CSCs in colonic tissue sections. We found that the number of GFP-positive CSCs at the crypt base was markedly reduced in Tsc2 CKO/Lgr5-GFP mice compared with control/Lgr5-GFP mice (Fig. 2a and Supplementary Fig. S2).

Wnt signaling is thought to play a key role in maintenance of ISCs and the proliferation of their progeny^5,10,22^. Reverse transcription (RT) and real-time polymerase chain reaction (PCR) analysis revealed that expression of the Wnt–β-catenin target genes for Lgr5, Ascl2, and c-Myc was significantly reduced, whereas that of the Wnt–β-catenin target genes for Axin2 and cyclin D1 tended to be reduced or was unaffected, respectively, in CECs isolated from the colon of Tsc2 CKO mice compared with those from control mice (Fig. 2b). These results thus suggested that ablation of Tsc2 in CECs leads to a reduction in the number of CSCs as a result of the suppression of Wnt–β-catenin signaling.

### Wnt–β-catenin signaling in the colon of Tsc2 CKO mice

**Figure 3:**
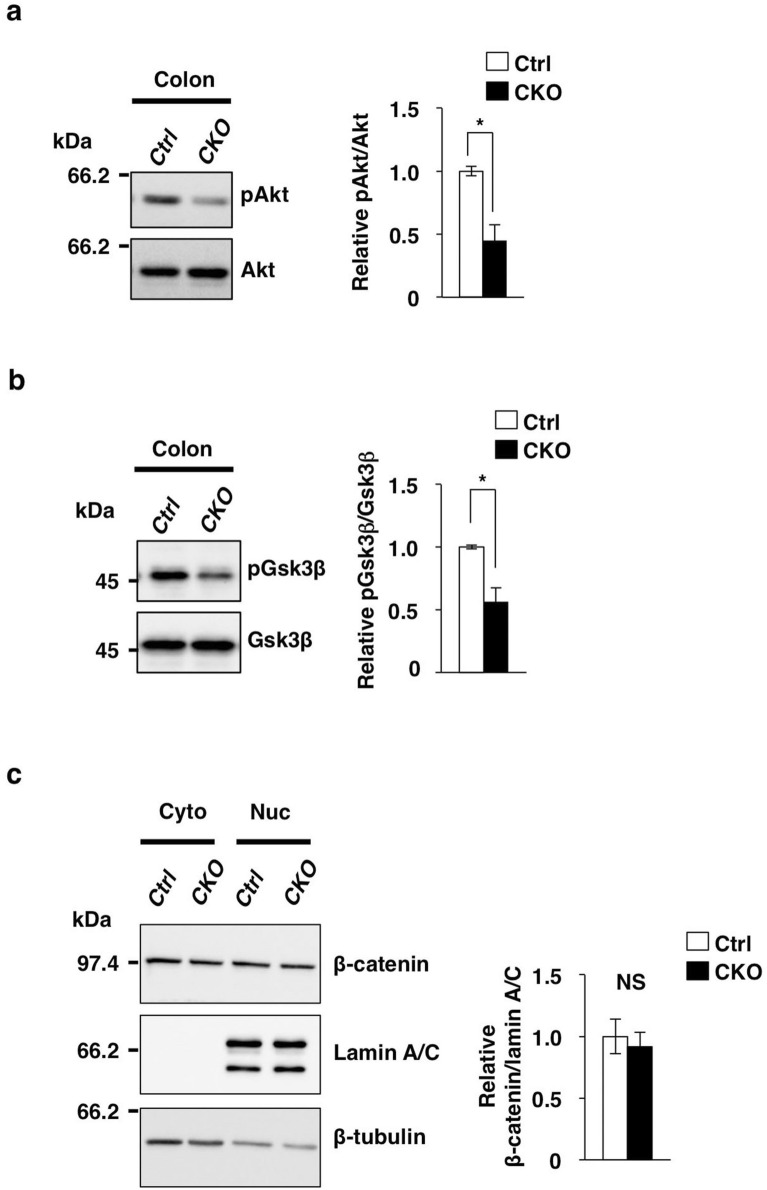
Wnt–β-catenin signaling in the colon of Tsc2 CKO mice. (**a**) Immunoblot analysis of lysates of the colon from control and Tsc2 CKO mice at 9- to 12-week-old with antibodies to phosphorylated (p) and total forms of Akt. Representative blots and densitometric analysis of the pAkt/Akt band intensity ratio are shown, with the quantitative data being expressed relative to the corresponding value for control mice and presented as means ± s.e. from three separate experiments. *P < 0.05 (Student’s *t* test). (**b**) Immunoblot analysis of lysates of the colon from control and Tsc2 CKO mice at 9- to 12-week-old with antibodies to phosphorylated (p) and total forms of Gsk3β. Representative blots and densitometric analysis of the pGsk3β/Gsk3β band intensity ratio are shown, with the quantitative data being expressed relative to the corresponding value for control mice and presented as means ± s.e. from three separate experiments. *P < 0.05 (Student’s *t* test). (**c**) Immunoblot analysis of cytoplasmic (Cyto) and nuclear (Nuc) fractions of the colon of control or Tsc2 CKO mice at 13- to 14-week-old with antibodies to β-catenin, to lamin A/C (nuclear marker), and to β-tubulin (cytoplasmic marker). Representative blots and densitometric analysis of the β-catenin to lamin A/C band intensity ratio are shown, with the quantitative data being expressed relative to the corresponding value for control mice and presented as means ± s.e. from three separate experiments. NS, not significant (Student’s *t* test). Uncropped immunoblot data of **(a)**, **(b)**, and **(c)** are shown in Supplementary Fig. S9.

We next investigated the molecular mechanism by which ablation of Tsc2 in CECs results in down-regulation of Wnt–β-catenin signaling. The binding of Wnt ligands to the Frizzled-LRP5/6 receptor complex results in suppression of glycogen synthase kinase 3β (Gsk3β), which is responsible for phosphorylation of β-catenin at serine-37 or serine-33 and its subsequent degradation by the ubiquitin–proteasome system^23^. The nonphosphorylated form of β-catenin thus accumulates, translocates to the nucleus, and acts as a transcriptional cofactor for T cell factor (Tcf) transcription factors, resulting in the transcription of Wnt–β-catenin target genes^24^. By contrast, the kinase Akt, an upstream activator of mTORC1, inhibits the activity of Gsk3β by mediating its phosphorylation at serine-9^25^. In melanocytes, feedback inhibition of Akt by aberrant activation of mTORC1 was shown to result in a reduction in the level of Gsk3β phosphorylation and down-regulation of Wnt target gene expression^26^. Indeed, we found that the amount of phosphorylated (activated) Akt was markedly decreased in the colon of Tsc2 CKO mice compared with that of control mice (Fig. 3a). In addition, phosphorylation of Gsk3β at serine-9 was attenuated in the colon of the mutant animals (Fig. 3b), suggesting that the activity of Gsk3β was increased. However, we found that the amount of β-catenin in the nuclear fraction of the colon was similar for both Tsc2 CKO and control mice (Fig. 3c). In addition, the amount of total β-catenin in the colon as well as the localization of β-catenin in CECs were similar for both Tsc2 CKO and control mice (Supplementary Fig. S3). The activation of mTORC1 has also been shown to suppress the amount of the Wnt receptor Frizzled at the cell surface, resulting in inhibition of Wnt signaling^27^. However, we found that the abundance of Frizzled 7 in CECs also did not differ between Tsc2 CKO and control mice (Supplementary Fig. S4). It was recently shown that the Ras-MEK-MAPK pathway suppresses Wnt ligand–induced gene expression by increasing the abundance of the ~ 50-kDa (shorter) isoforms of Tcf4 (Tcf M/S), which are thought to be transcriptionally inactive and to inhibit activation of Wnt target genes^9^. However, we again found that the amount of the Tcf4 M/S isoforms was not increased in the colon of Tsc2 CKO mice compared with that of control mice (Supplementary Fig. S5). The abundance of the ~ 70-kDa (longer) isoform of Tcf4 (Tcf4 E) in the colon was also similar for the two genotypes (Supplementary Fig. S5).

### Promotion of the development of colonic organoids by Tsc2 ablation in CECs

**Figure 4:**
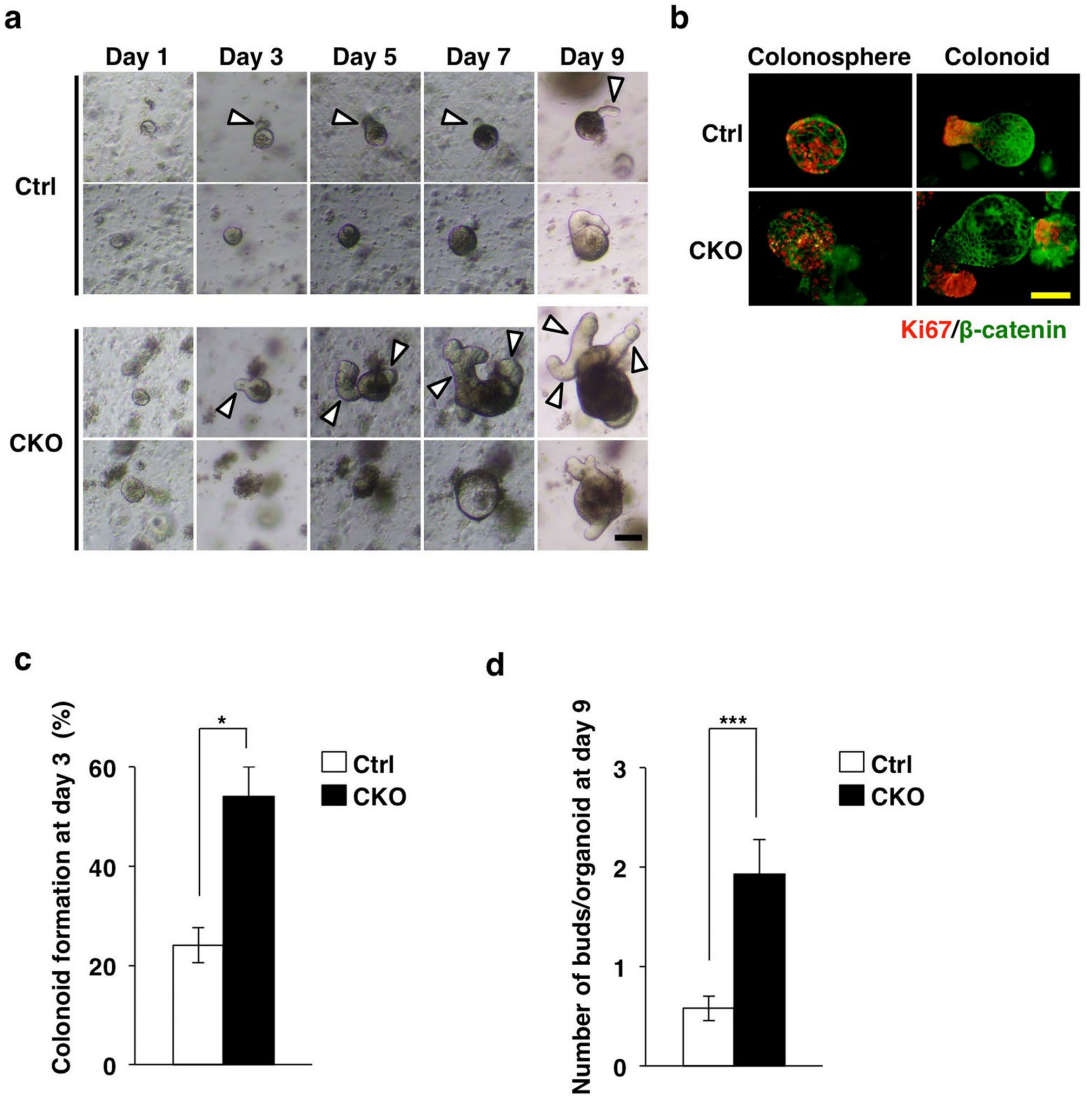
Promotion of the development of colonic organoids by Tsc2 ablation. (**a**) Representative phase-contrast images of colonic organoids derived from the colon of 9- to 13-week-old control or Tsc2 CKO mice at 1, 3, 5, 7, and 9 days after cell plating. The white arrowheads indicate cryptlike buds. Scale bar, 100 μm. (**b**) Immunofluorescence analysis of Ki67 (red) and β-catenin (green) in colonic organoids cultured for 7 days as in **(a)**. Scale bar, 100 μm. (**c**) The number of colonoids was determined as a percentage of colonic organoids at 3 days after cell plating as in **(a)**. Data are means ± s.e. for a total of 105 (control) or 119 (Tsc2 CKO) organoids in three independent experiments. *P < 0.05 (Student’s *t* test). (**d**) The number of buds per colonic organoid was determined at 9 days after cell plating. Data are means ± s.e. for a total of 38 (control) or 28 (Tsc2 CKO) organoids in three independent experiments. ***P < 0.001 (Student’s *t* test).

Given that we found that the proliferation of CECs was markedly increased in Tsc2 CKO mice, we next examined the development of colonic epithelial organoids prepared from the mutant and control animals. Intestinal organoids constitute three-dimensional “mini-guts” and are thought to mimic the proliferation and differentiation of IECs *in vivo*^28^. Colonic organoids were allowed to develop under a culture condition similar to that used for small intestinal organoids, with the exception that Wnt-3A and nicotinamide were added to the culture medium^29,30^. One day after seeding of cells from control or Tsc2 CKO mice, the development of spherical structures known as colonospheres^31^ was apparent (Fig. 4a). At 3 days after cell seeding, some colonospheres had generated multiple budlike protrusions that are thought to mimic colonic crypts, with the resulting structures being known as colonoids^31^ (Fig. 4a). The expression of Ki67 was localized to the buds of colonoids derived from either control or Tsc2 CKO mice (Fig. 4b), suggesting that these buds do indeed represent a cryptlike structure harboring CSCs or TA cells. The percentage of colonoids among total colonic organoids for Tsc2 CKO mice at 3 days after cell seeding was markedly higher than that apparent for the control (Fig. 4c). Consistent with this finding, the number of buds formed per organoid was also higher for Tsc2 CKO mice than for control mice (Fig. 4d). These results thus suggested that Tsc2 ablation in CECs accelerates the development of colonoids in organoid culture.

### Increased susceptibility of Tsc2 CKO mice to DSS-induced colitis

**Figure 5:**
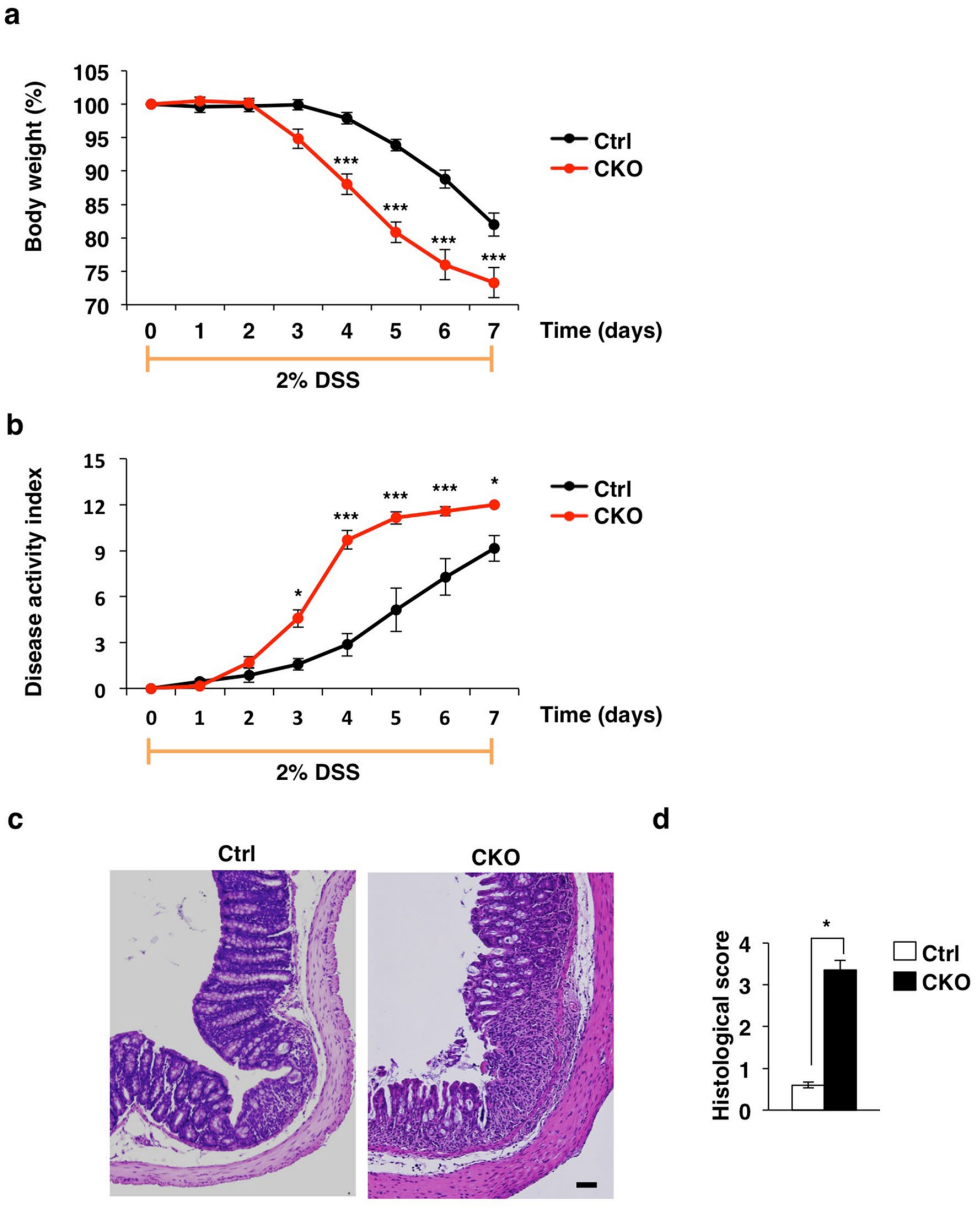
Increased susceptibility of Tsc2 CKO mice to to DSS-induced colitis. (**a**) Body weight of 9- to 11-week-old control (*n* = 6) and Tsc2 CKO (*n* = 6) mice supplied with drinking water containing 2% DSS for 7 days. Data are expressed as a percentage on the initial value and are means ± s.e. ***P < 0.001 versus the corresponding value for control mice (two-way repeated ANOVA and Sidak’s test). (**b**) Disease activity index for control (*n* = 6) and Tsc2 CKO (*n* = 6) mice treated as in **(a)**. Data are means ± s.e.. *P < 0.05; ***P < 0.001 versus the corresponding value for control mice (two-way repeated ANOVA and Sidak’s test). (**c**) Representative images of hematoxylin-eosin–stained, paraffin-embedded colon sections from control and Tsc2 CKO mice at 3 days after the start of DSS treatment as in **(a)**. Scale bar, 100 μm. (**d**) Histological score of 9- to 11-week-old control (*n* = 4) and Tsc2 CKO (*n* = 4) mice determined from sections similar to those in **(c)**. Data are means ± s.e.. *P < 0.05 (Student’s *t* test).

Dextran sulfate sodium (DSS)–induced colitis is studied as a model of human inflammatory bowel diseases as well as of regeneration of the colonic epithelium in response to external stress^32–34^. We therefore tested the susceptibility of Tsc2 CKO mice to DSS-induced colitis. Tsc2 CKO and control mice were supplied with drinking water containing 2% DSS for 7 days. After administration of DSS for 3 days, Tsc2 CKO mice showed a marked loss of body weight and an increased disease activity index compared with control mice (Fig. 5a, b). At 3 days after the onset of DSS treatment, hematoxylin–eosin staining of the colon also showed that Tsc2 CKO mice developed more severe colitis compared with control mice (Fig. 5c, d). Collectively, these results indicated that excessive activity of mTORC1 induced by Tsc2 ablation in CECs increases the susceptibility of mice to DSS-induced colitis.

## Discussion

**Figure 6:**
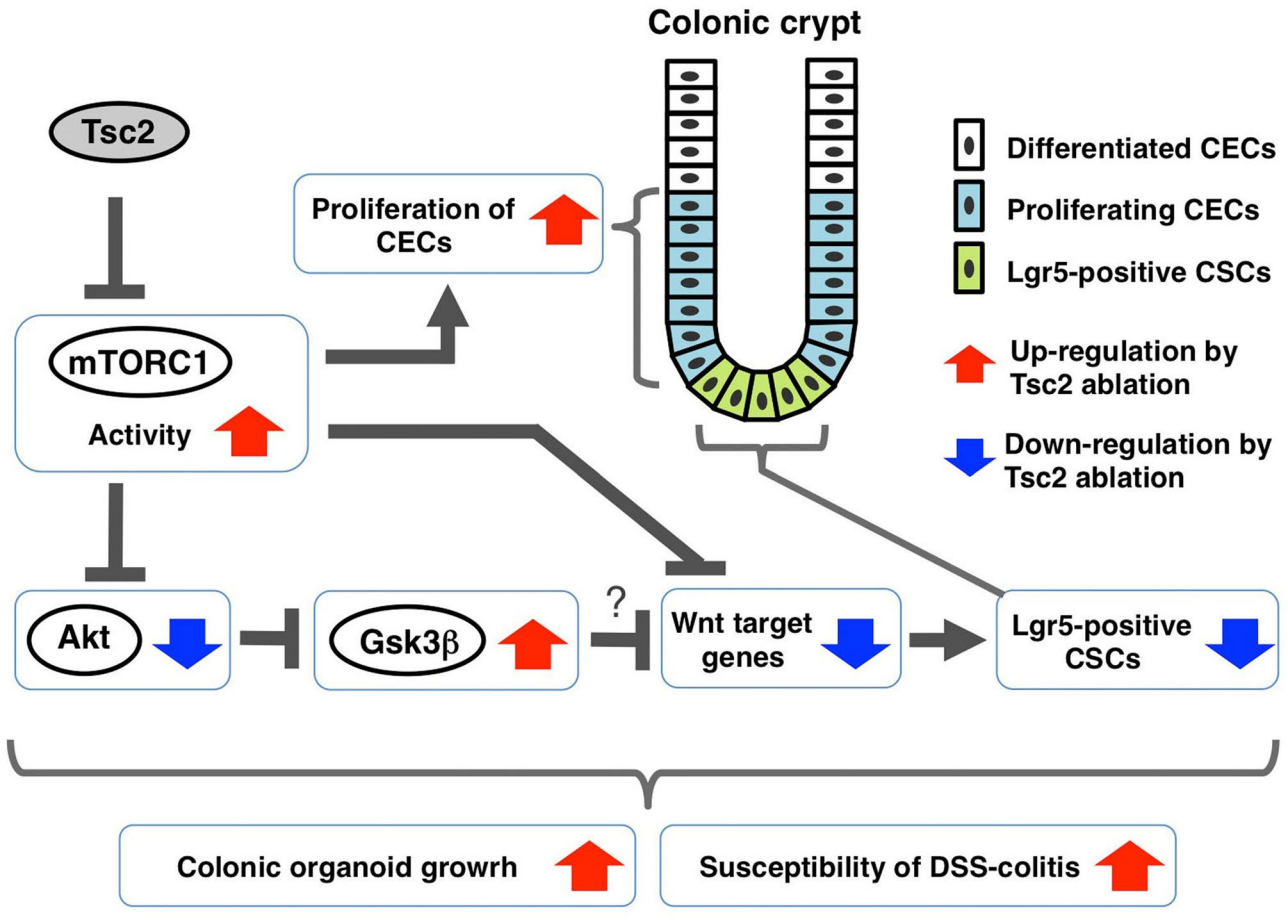
Role of mTORC1 in the homeostatic regulation of CECs. The activity of mTORC1 is likely important for promotion of the proliferative activity of CEC, particularly a proliferating progenitor of differentiated CECs, such as TA cells, in the crypt bottom portion. The activity of mTORC1 is also important for negative regulation of the population of Lgr5-positive colonic stem cells and the expression of Wnt target genes, in part through regulation of Akt and Gsk3β, in CECs. Thus, the mTORC1 activity likely participates in development of colonic organoid and the susceptibility of DSS-colitis, contributing to the homeostasis of CECs.

We have here shown that the proliferative activity of CECs is markedly increased in the colonic epithelium of Tsc2 CKO mice. In particular, mTORC1 is likely important for promotion of the proliferative activity of the CEC progenitor, such as TA cells, in the crypt bottom (Fig. 6). Moreover, the development of colonic organoids—in particular, the formation of cryptlike structures—was promoted by Tsc2 ablation in CECs. Given that mTORC1 acts downstream of receptors for growth factors such as epidermal growth factor, our results thus suggest that mTORC1 plays an important role in regulation of the proliferative activity of CECs. Ras and Src family kinases, both of which also function downstream of growth factor receptors, were previously shown to promote not only the proliferation of IECs but also the generation and differentiation of secretory cells such as goblet cells^6–9^. However, we found that the number of Muc2-positive mucus-secreting cells in the colon did not differ between control and Tsc2 CKO mice, suggesting that mTORC1 activity is not essential for differentiation of these cells from their progenitors. We also found that there was no marked difference in epithelial cell morphology between control and Tsc2 CKO mice. Thus, the Tsc2 ablation likely has a minimal effect on the differentiated CECs. Further investigation is necessary to clarify whether differentiated CECs of Tsc2 CKO mice were functionally normal, however.

We found that ablation of Tsc2 in CECs reduced the population of Lgr5-positive CSCs as well as the expression of Wnt target genes in the colon, suggesting that the activation of mTORC1 counter-regulates Wnt signaling in the colonic epithelium. The amount of Wnt target genes could be also reduced in the remaining Lgr5-positive cells in Tsc2 CKO mice. This notion is consistent with the previous observation that Tsc1/2 is required for maintenance of ISCs in *Drosophila*^35^. In addition, the activity of mTORC1 was recently shown to be important for suppression of Wnt signaling in melanocytes^26^ as well as in IECs of the small intestine^27^. The molecular mechanism of such inhibition of Wnt signaling by mTORC1 remains unclear, however. In melanocytes, feedback inhibition of Akt by aberrant activation of mTORC1 likely results in a reduced level of phosphorylation at serine-9 and in consequent activation of Gsk3β, leading to down-regulation of Wnt target gene expression^26^. Indeed, we found that phosphorylation of both Akt and Gsk3β was markedly decreased in the colon of Tsc2 CKO mice. An increase in the activity of Gsk3β may therefore promote β-catenin degradation and consequent down-regulation of Wnt target gene expression. However, we did not detect a difference in the amount of β-catenin in the nuclear fraction of total colon tissue between Tsc2 CKO and control mice. Further investigation using isolated Lgr5-positive CSCs will be required to evaluate the amount of β-catenin in the nuclear fraction of CSCs from Tsc2 CKO mice. The proliferative activity of CECs, particularly in the bottom portion of the colonic crypts, was markedly increased, while the Lgr5-positive CSCs was reduced in Tsc2 CKO mice. It is likely that the remaining Lgr5-positive stem cells, or an alternate population, which responds differently to mTORC1 activation, might participate in generation of the CEC progenitor, such as TA cells, that manifested increased proliferative activity in the crypt bottom of the Tsc2 CKO mice.

Finally, we found that Tsc2 CKO mice manifested an increased susceptibility to DSS-induced colitis. Suppression of mTORC1 activity with either an mTORC1 inhibitor or genetic ablation of Rheb was also previously shown to increase the susceptibility of mice to DSS-induced colitis^36^. Furthermore, mTORC1-mediated activation of signal transducer and activator of transcription 3 (Stat3) induced by interleukin-6 was found to be important for IEC proliferation and tissue regeneration in the intestine^36^. Indeed, we have here shown that the proliferative activity of CECs is increased in Tsc2 CKO mice. The increased susceptibility of Tsc2 CKO mice to DSS-induced colitis might thus be attributable to suppression of Wnt signaling and the reduced number of CSCs in these animals. Moreover, mTORC1 activity is an important suppressor of the induction of autophagy^39^, and we found that the LC3II/LC3I protein ratio, a marker of autophagic activity, tended to be reduced in CECs of Tsc2 CKO mice compared with those of control mice (Supplementary Fig. S6). Given that proper regulation of autophagy is thought to be important for protection against colitis in humans and mice^40,41^, the increased susceptibility of Tsc2 CKO mice to DSS-induced colitis also might be attributable to dysregulation of autophagy. Lgr5-positive ISCs have been thought to be essential for regeneration of the intestine after injury^37^. A recent study has demonstrated that revival stem cells are expanded by intestinal damage in a manner dependent of a transcription factor, Yap1^38^. These cells are thought to reconstitute Lgr5-positive ISCs and are important for regeneration of the intestine after injury. Ablation of Tsc2 might therefore affect the expansion of revival stem cells after DSS treatment, whereas the abundance of Yap1 in the colon of Tsc2 CKO mice was not changed compared with that for control mice before or after DSS treatment (Supplementary Fig. S7). Further investigation is necessary to clarify the molecular mechanisms by which mTORC1 regulates CEC homeostasis after tissue damage.

## Methods

### Antibodies and reagents

Mouse monoclonal antibodies (mAbs) to β-catenin and to β-tubulin were obtained from BD Biosciences (San Diego, CA) and Sigma-Aldrich (St. Louis, MO), respectively. A rat mAb to BrdU and rabbit polyclonal antibodies (pAbs) to Frizzled 7 were from Abcam (Cambridge, MA). Rabbit pAbs to Ki67 were obtained from Acris (Herford, Germany), and those to Muc2 were from Santa Cruz Biotechnology (Santa Cruz, CA). Rabbit mAbs or pAbs to S6, to phosphorylated S6 (Ser235/236), to 4E-BP1, to phosphorylated 4E-BP1 (Thr37/46), to Tsc2, to Tcf4, to Akt, to phosphorylated Akt (Ser473), to Gsk3β, to phosphorylated Gsk3β (Ser9), to lamin A/C, to Yap1, and to LC3 were obtained from Cell Signaling Technology (Beverly, MA). Horseradish peroxidase– or Cy3–conjugated secondary antibodies were obtained from Jackson ImmunoResearch (West Grove, PA). Alexa Fluor 488–conjugated secondary antibodies were obtained from ThermoFisher (Waltham, MA). 4′,6-diamidino-2-phenylindole (DAPI) was obtained from Nacalai Tesque (Kyoto, Japan). BrdU was obtained from Sigma-Aldrich. Mayer’s hemalum solution was obtained from Merck KGaA (Darmstadt, Germany). Eosin was obtained from Wako (Osaka, Japan).

### Mice

For generation of *Tsc2*^fl/+^;*Villin-Cre* mice, *Villin-Cre* mice^17^ were crossed with *Tsc2*^fl/fl^ mice^42^. The resulting *Tsc2*^fl/+^;*Villin-Cre* offspring were crossed with *Tsc2*^fl/fl^ mice to obtain *Tsc2*^fl/fl^;*Villin-Cre* (Tsc2 CKO) and *Tsc2*^fl/fl^ (control) mice. For generation of *Tsc2*^fl/+^;*Villin-Cre*;*Lgr5-Gfp-CreERT2* mice, *Tsc2*^fl/fl^;*Villin-Cre* mice were crossed with *Lgr5-Gfp-CreERT2* mice^21^. The resulting *Tsc2*^fl/+^;*Villin-Cre*;*Lgr5-Gfp-CreERT2* offspring were crossed with *Tsc2*^fl/fl^ mice to obtain *Tsc2*^fl/fl^;*Villin-Cre*;*Lgr5-Gfp-CreERT2* (Tsc2 CKO/Lgr5-GFP) mice and *Tsc2*^fl/fl^;*Lgr5-Gfp-CreERT2* (control/Lgr5-GFP) mice. All mice were maintained at the Institute for Experimental Animals at Kobe University Graduate School of Medicine under the specific pathogen–free condition. Mice were given normal chow (CLEA Rodent diet CE-2, CLEA Japan, Tokyo, Japan) and water ad libitum. This study was approved by the Institutional Animal Care and Use Committee of Kobe University (permit numbers P170707, P150506, and P120508-R2), and all animal experiments were performed according to Kobe University Animal Experimentation Regulations.

### Histology and immunohistofluorescence analysis

For histological analysis, the colon was removed and immediately fixed with 4% paraformaldehyde in phosphate-buffered saline (PBS) for > 12 h at room temperature. Paraffin-embedded sections with a thickness of 6 μm were then prepared and stained with hematoxylin–eosin. For immunohistofluorescence analysis, the colon was removed and fixed immediately for 3 h at room temperature with 4% paraformaldehyde in PBS. The tissue was then incubated overnight in PBS containing 30% (w/v) sucrose, embedded in optimal cutting temperature (OCT) compound (Sakura, Tokyo, Japan), and rapidly frozen in liquid nitrogen. Frozen sections with a thickness of 5 μm were prepared with a cryostat, mounted on glass slides, air-dried, and subjected to immunofluorescence staining with primary antibodies and fluorescent dye–labeled secondary antibodies with or without DAPI as described previously^8,43^. Fluorescence images were obtained with a fluorescence microscope (BX51; Olympus, Tokyo, Japan).

### BrdU incorporation assay

Mice were injected intraperitoneally with BrdU (10 mg per kilogram of body weight). After 2 h, frozen sections of the colon were prepared as described above and were immunostained as described previously^16^. Fluorescence images were obtained with a fluorescence microscope (BX51, Olympus).

### Isolation of mouse CECs

Mouse CECs were isolated as previously described but with a slight modification^44^. In brief, the freshly isolated colon was washed with PBS, cut into small pieces, washed three times with Hanks’ balanced salt solution (HBSS) containing 1% fetal bovine serum and 25 mM HEPES–NaOH (pH 7.5), and then incubated three times on a rolling platform for 15 min at room temperature in HBSS containing 5 mM EDTA and 25 mM HEPES–NaOH (pH 7.5). After removal of tissue debris, CECs were isolated by centrifugation at 250 × *g* for 10 min at 4 °C and washed three times with PBS.

### Preparation of cytoplasmic and nuclear extracts

Cytoplasmic and nuclear extracts were prepared from the colon of mice with the use of the NE-PER reagent (Pierce, Rockford, IL).

### Immunoblot analysis

Isolated CECs or tissue was lysed with a lysis solution containing 20 mM Tris–HCl (pH 7.5), 150 mM NaCl, 2 mM EDTA, 1% Nonidet P-40, 1% sodium deoxycholate, 0.1% sodium dodecyl sulfate, 50 mM NaF, 1 mM sodium vanadate, and 1% protease inhibitor cocktail (Nacalai Tesque). The lysates were centrifuged at 17,500 × *g* for 15 min at 4 °C, and the resulting supernatants were subjected to immunoblot analysis as previously described^43,44^.

### RT and real-time PCR analysis

Isolation of total RNA and quantitative RT-PCR analysis were performed as described previously^45^, with minor modifications. In brief, total RNA was prepared from isolated CECs with the use of Sepasol RNA I (Nacalai Tesque) and an RNeasy Mini Kit (Qiagen, Hilden, Germany). First-strand cDNA was synthesized from portions (0.8 μg) of the RNA with the use of a QuantiTect Reverse Transcription Kit (Qiagen). The cDNA fragments of interest were amplified by PCR with the use of Fast Start SYBR Green Master (Roche, Penzberg, Germany) and a LightCycler 480 instrument (Roche). The amplification was analyzed with the use of LightCycler 480 software (Roche). The abundance of each target mRNA was normalized by that of hypoxanthine–guanine phosphoribosyltransferase 1 (Hprt1) mRNA. Primer sequences (forward and reverse, respectively) were as follows: Hprt1, 5′-CAGTCCCAGCGTCGTGATTA-3′ and 5′-GGCCTCCCATCTCCTTCATG-3′; Lgr5, 5′-ACCCGCCAGTCTCCTACATC-3′ and 5′-GCATCTAGGCGCAGGGATTG-3′; Ascl2, 5′-CTACTCGTCGGAGGAAAG-3′ and 5′-ACTAGACAGCATGGGTAAG-3′; c-Myc, 5′-CTGGATTTCCTTTGGGCGT-3′ and 5′-TGGTGAAGTTCACGTTGAGGG-3′; Axin2, 5′-GGACTGGGGAGCCTAAAGGT-3′ and 5′-AAGGAGGGACTCCATCTACGC-3′; and cyclin D1, 5′-CAGACGTTCAGAACCAGATTC-3′ and 5′-CCCTCCAATAGCAGCGAAAAC-3'.

### Colonic organoid culture

Colonic organoid culture was performed as previously described^29,30^ but with a slight modification. In brief, the removed colon from mice was opened longitudinally and incubated in PBS containing 5 mM EDTA for 30 min at 4 °C. The colon was then incubated in Dulbecco’s modified Eagle’s medium–F12 (Invitrogen, Carlsbad, CA) containing penicillin–streptomycin (100 U/ml) (Invitrogen), 10 mM HEPES (Invitrogen), and collagenase type IV (500 U/ml) (Worthington Biochemical, Lakewood, NJ) for 30 min at 37 °C to isolate colonic crypts. The isolated crypts were mixed with Matrigel (BD Biosciences) and transferred to 48-well plates. After polymerization of the Matrigel, advanced Dulbecco’s modified Eagle’s medium–F12 (Invitrogen) supplemented with penicillin–streptomycin (100 U/ml), 10 mM HEPES, 1 × GlutaMAX (Invitrogen), 1 × N2 (Invitrogen), 1 × B27 (Invitrogen), 1.25 mM *N*-acetylcysteine (Sigma-Aldrich), 10% R-spondin1–Fc–conditioned medium, epidermal growth factor (50 ng**/**ml) (Peprotech, Rocky Hill, NJ)**,** Noggin (100 ng/ml) (Peprotech), Wnt-3A (100 ng/ml) (Merck KGaA), and 10 mM nicotinamide (Sigma-Aldrich) was overlaid on the gel in each well.

### Immunofluorescence analysis of colonic organoids

Whole-mount immunofluorescence analysis was performed as described previously, but with a slight modification^46^. In brief, colonic organoids in Matrigel were fixed with 4% paraformaldehyde for 30 min at room temperature and isolated from the gel with Cell Recovery Solution (BD Biosciences). The isolated organoids were then incubated at room temperature first with antibodies to Ki67 and to β-catenin for 3 h and then with fluorescent dye–labeled secondary antibodies for 1 h. Fluorescence images were obtained with a fluorescence microscope (BX51, Olympus).

### DSS-induced colitis

Mice were treated with 2% DSS (molecular mass of 36 to 50 kDa) (MP Biomedicals, Santa Ana, CA) in drinking water for 7 days. Mice were monitored daily for weight loss, stool consistency, and blood in the stool, and disease activity was scored as described previously^47,48^. Weight loss was scored as follows: 0 =  < 1%, 1 = 1% to 5%, 2 = 5% to 10%, 3 = 10% to 20%, and 4 =  > 20%. Stool consistency was scored as: 0 = normal, 2 = loose stools, and 4 = liquid stools. Blood in the stool was scored as: 0 = no blood as revealed with the use of the guaiac occult blood test (ColoScreen-ES; Helena, Beaumont, TX), 2 = positive occult blood test, and 4 = gross bleeding. The total score for weight loss, diarrhea, and blood in the stool, ranging from 0 (normal) to 12 (severe), was determined as the disease activity index. For scoring of colonic inflammation by histologic examination, a combined score for inflammatory cell infiltration, tissue damage, and crypt structure was determined in a blinded manner as described previously^45^. Inflammatory cell infiltration was scored as follows: 0 = the presence of occasional inflammatory cells in the lamina propria, 1 = the presence of an increased number of inflammatory cells in the lamina propria, 2 = confluence of inflammatory cells extending into the submucosa, and 3 = transmural extension of the infiltrate. Tissue damage was scored as: 0 = no mucosal damage, 1 = lymphoepithelial lesions, 2 = surface mucosal erosion, and 3 = extensive mucosal damage and extension into deeper structures of the colonic wall. Crypt structure was scored as: 0 = normal structure, 1 = occasional hyperplasia without depletion of goblet cells, 2 = hyperplasia with depletion of goblet cells, and 3 = distortion of crypts and the presence of crypt abscesses. Each section was assigned a score based on the three criteria, ranging from 0 (no change) to 9 (severe).

### Statistical analysis

Data are presented as means ± s.e. and were analyzed with Student’s *t* test, or by two-way repeated analysis of variance (ANOVA) followed by Sidak’s test, with the use of GraphPad Prism software version 6.0 (GraphPad, San Diego, CA) as described previously^48^. A* P* value of < 0.05 was considered statistically significant.

## Supplementary information

Supplementary Information.

## Data Availability

The authors declare that all other data supporting the findings of this study are available within the article and its Supplementary Information Files.
