# Supplementary material for: Regulation of colonic epithelial cell homeostasis by mTORC1
Source: Sci Rep. 2020 Aug 14;10:13810. doi: 10.1038/s41598-020-70655-1 (PMC7427982; doi:10.1038/s41598-020-70655-1)
Supplement: Supplementary file 1 — Supplementary Information. [file 41598_2020_70655_MOESM1_ESM.pdf]

Supplementary Information

Regulation of Colonic Epithelial Cell Homeostasis by mTORC1

Takenori Kotani[1], Jajar Setiawan[1,2], Tasuku Konno[1], Noriko Ihara[1], Saki Okamoto[1], Yasuyuki Saito[1], Yoji Murata[1], Tetsuo Noda[3], and Takashi Matozaki[1,*]

[1]Division of Molecular and Cellular Signaling, Department of Biochemistry and Molecular Biology, Kobe University Graduate School of Medicine, Kobe, Japan
[2]Department of Physiology, Faculty of Medicine, Public Health, and Nursing, Universitas Gadjah Mada, Yogyakarta, Indonesia
[3]Department of Cell Biology, Cancer Institute, Japanese Foundation for Cancer Research, Tokyo, Japan.
*Corresponding author

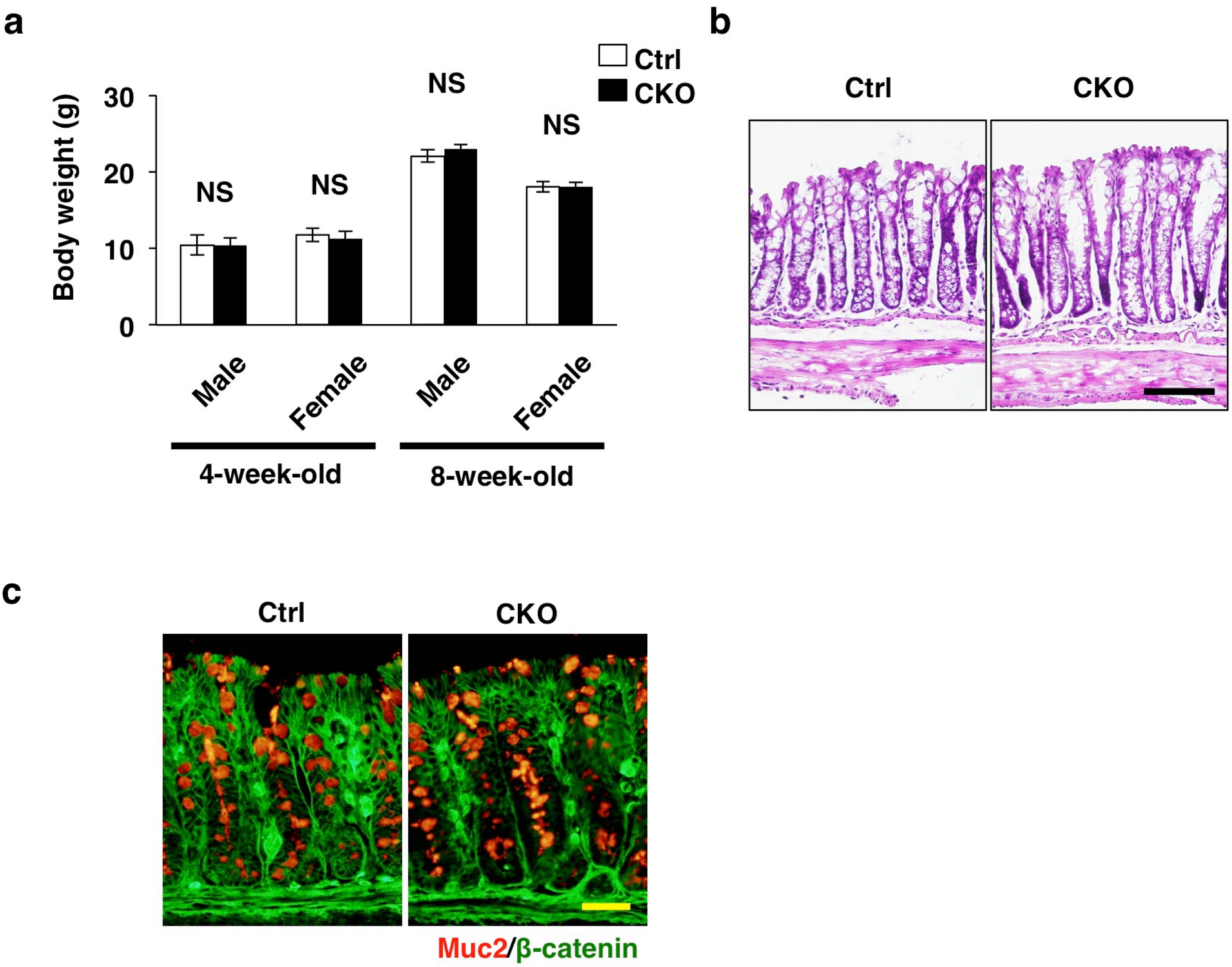

Supplementary Figure S1. Colonic structure of control and Tsc2 CKO mice. (**a**) Body weight of 4- or 8-week-old control (male, n = 6; female, n = 5) or Tsc2 CKO (male, n = 6; female, n = 5) mice. NS, not significant (Student's t test). (**b**) Hematoxylin-eosin staining of paraffin-embedded sections of the colon from 16-week-old control or Tsc2 CKO mice. Scale bar, 100 μm. (**c**) Immunohistofluorescence analysis of frozen sections of the colon from 8-week-old control or Tsc2 CKO mice with antibodies to Muc2 (red) and to β-catenin (green). Scale bar, 100 μm.

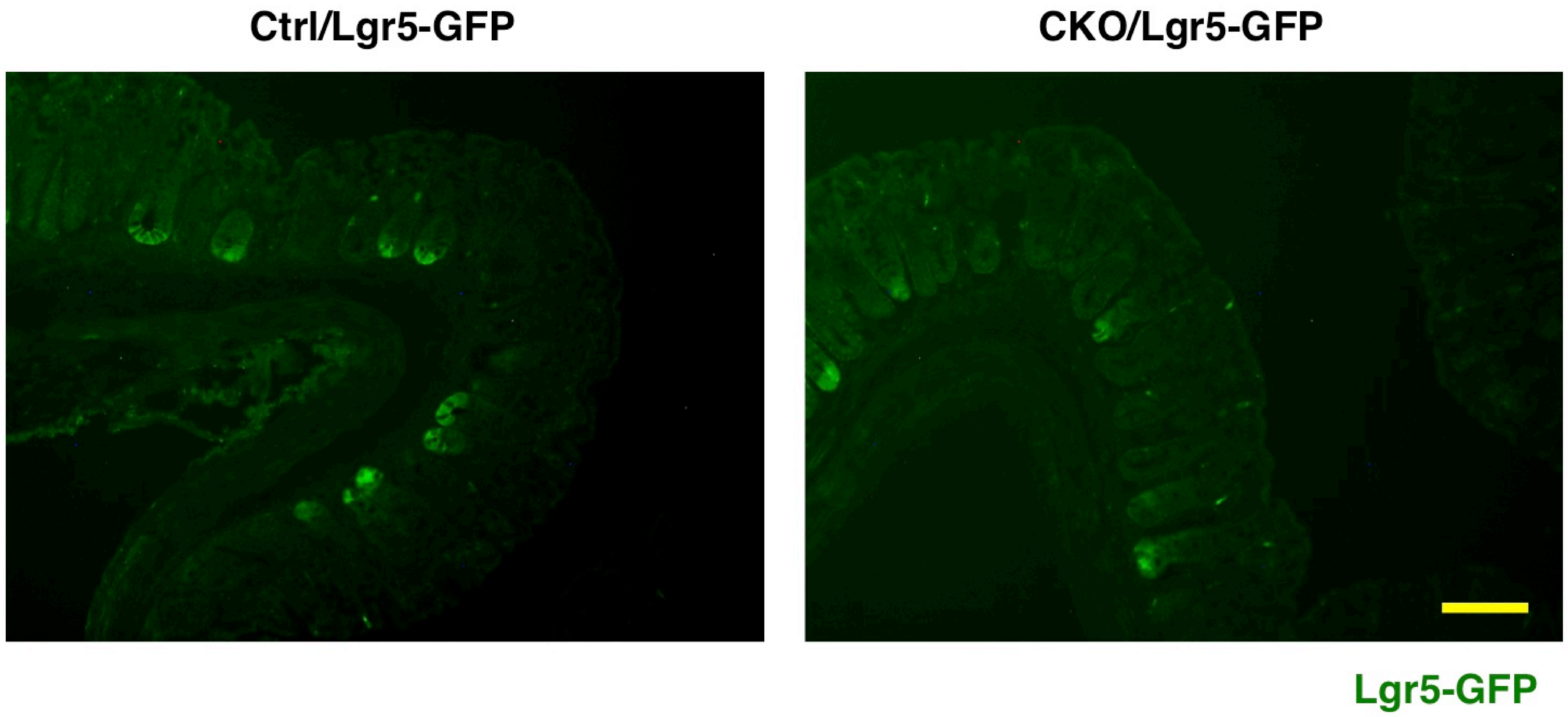

Supplementary Figure S2. Marked reduction in the number of Lgr5-positive crypts in the colon of Tsc2 CKO mice. Representative images of colon sections from control/Lgr5-GFP and Tsc2 CKO/Lgr5-GFP mice are shown. GFP fluorescence is green. Scale bar, 100 µm.

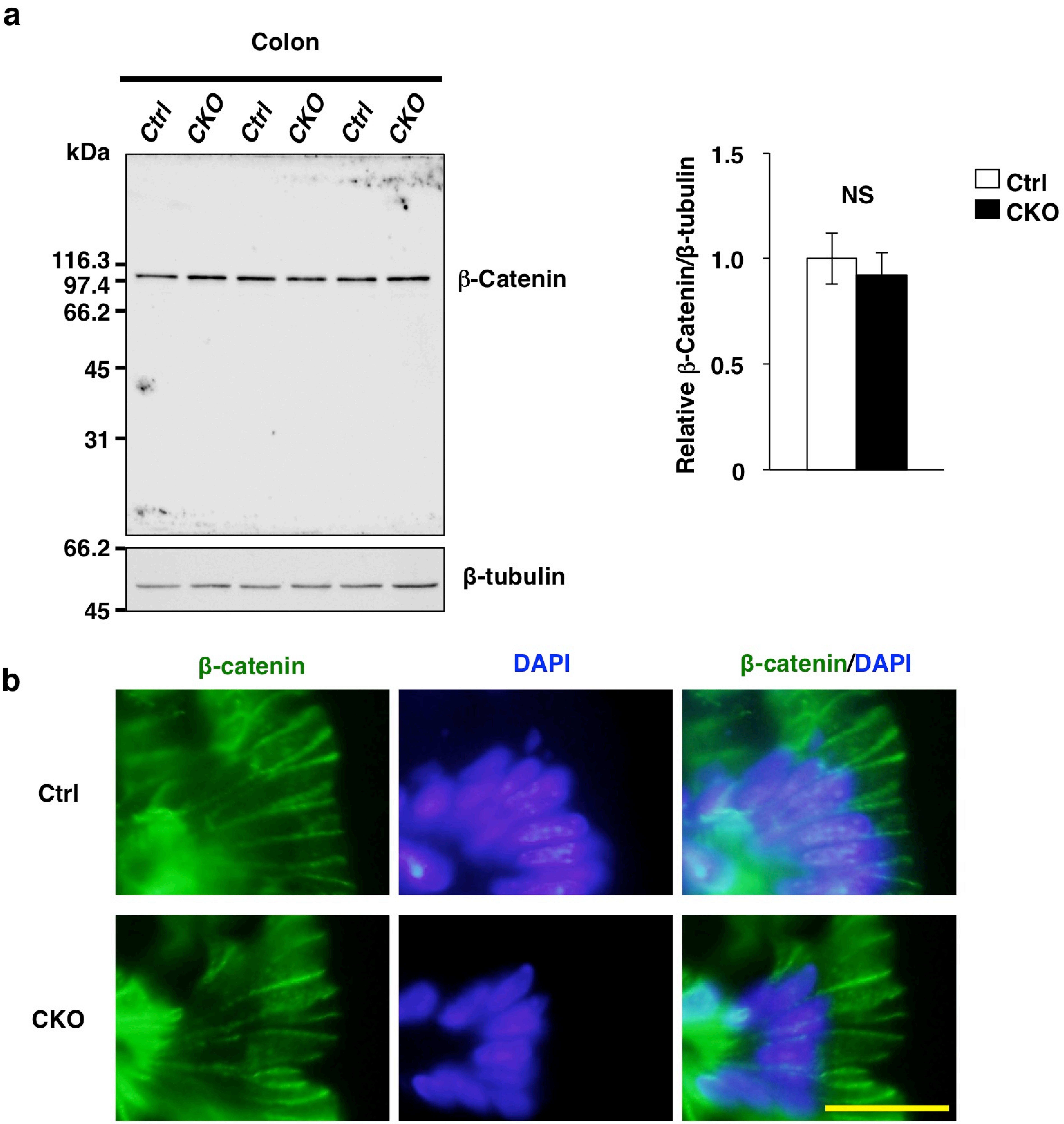

Supplementary Figure S3. Expression and localization of β-catenin in the colon of control and Tsc2 CKO mice. (**a**) Lysates of the colon from control and Tsc2 CKO mice at 13- to 14-week-old were subjected to immunoblot analysis with antibodies to β-catenin and to β-tubulin . Representative blots as well as densitometric analysis of the β-catenin/β-tubulin band intensity ratio are shown, with the quantitative data being expressed relative to the corresponding value for control mice and presented as means ± s.e. from three separate experiments. NS, not significant (Student's *t* test). (**b**) Frozen sections of the colon from control or Tsc2 CKO mice were stained with antibodies to β-catenin (green) and DAPI (blue). Scale bar, 20 μm.

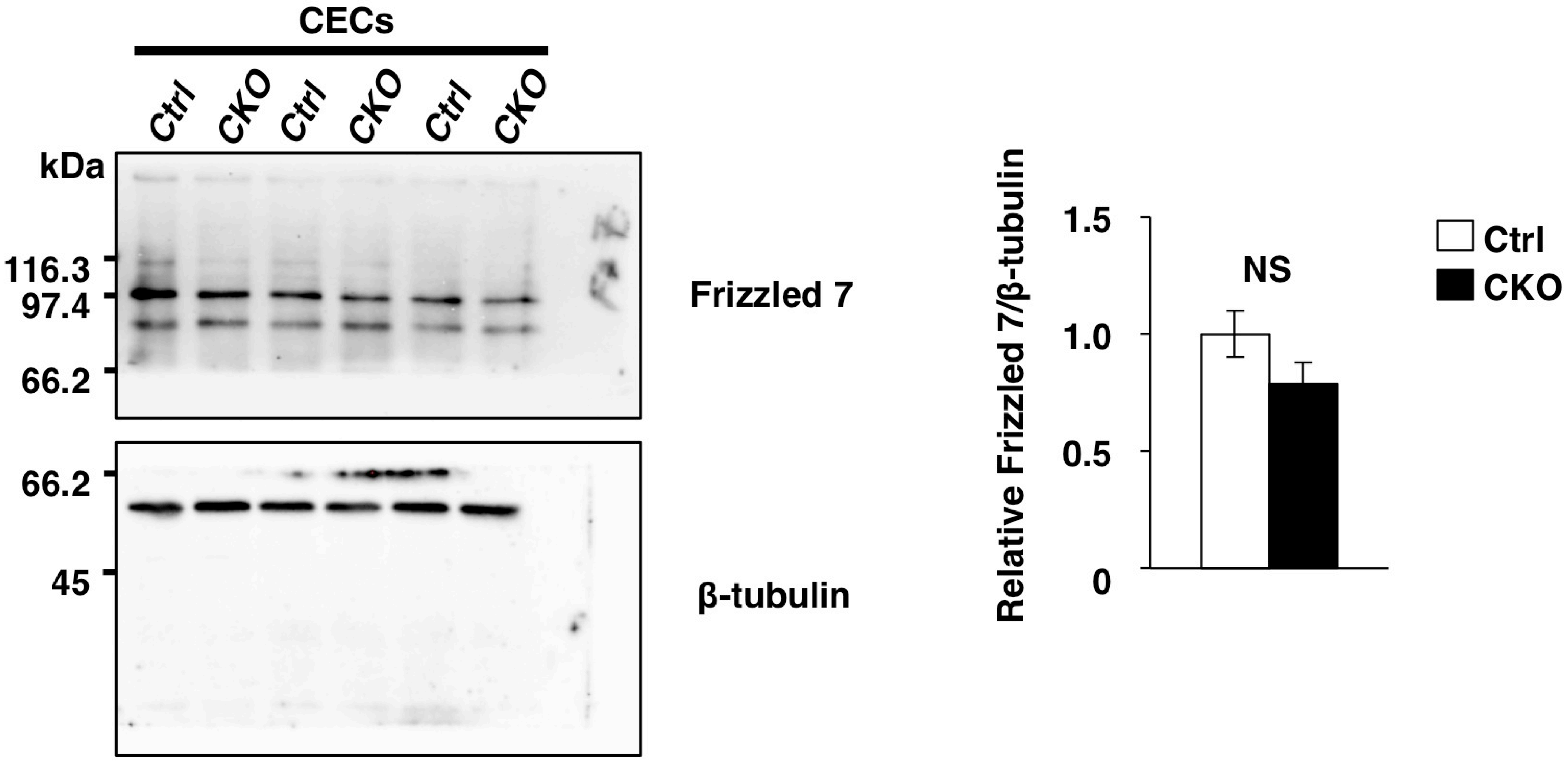

Supplementary Figure S4. Expression of Frizzled 7 in CECs of control and Tsc2 CKO mice. Lysates of CECs from control and Tsc2 CKO mice at 9- to 12-week-old were subjected to immunoblot analysis with antibodies to Frizzled 7 and to β-tubulin. Representative blots as well as densitometric analysis of the Frizzled 7/β-tubulin band intensity ratio are shown, with the quantitative data being expressed relative to the corresponding value for control mice and presented as means ± s.e. from three separate experiments. NS, not significant (Student's *t* test).

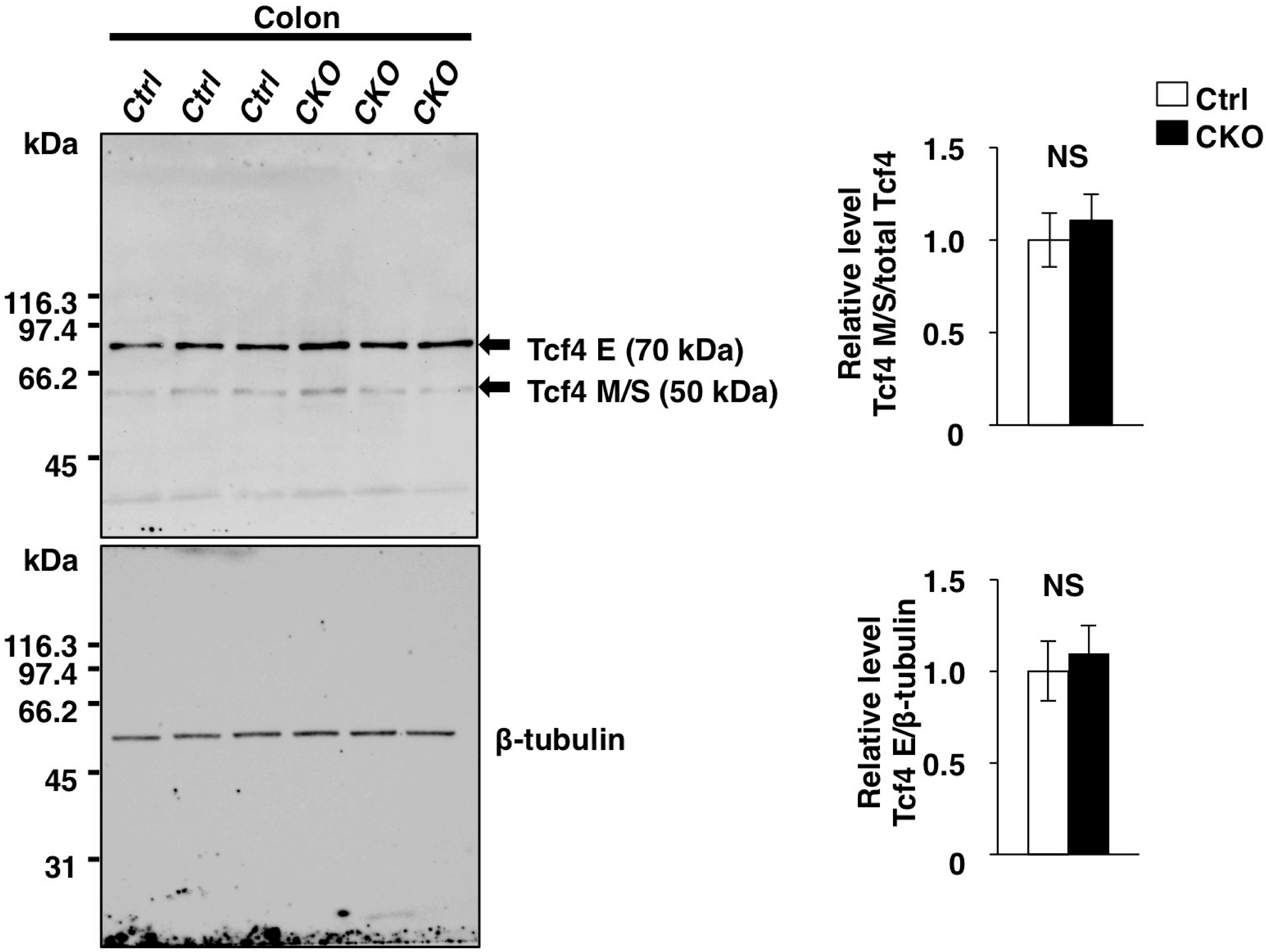

Supplementary Figure S5. Expression of Tcf4 in the colon of control and Tsc2 CKO mice. Lysates of the colon from control and Tsc2 CKO mice at 13- to 14-week-old were subjected to immunoblot analysis with antibodies to Tcf4 and to β-tubulin. Representative blots as well as densitometric analysis of the ratio of the Tcf4 M/S isoforms to total Tcf4 or of the Tcf4 E isoform to β-tubulin are shown. Quantitative data are expressed relative to the corresponding value for control mice and are means ± s.e. from three separate experiments. NS, not significant (Student's *t* test).

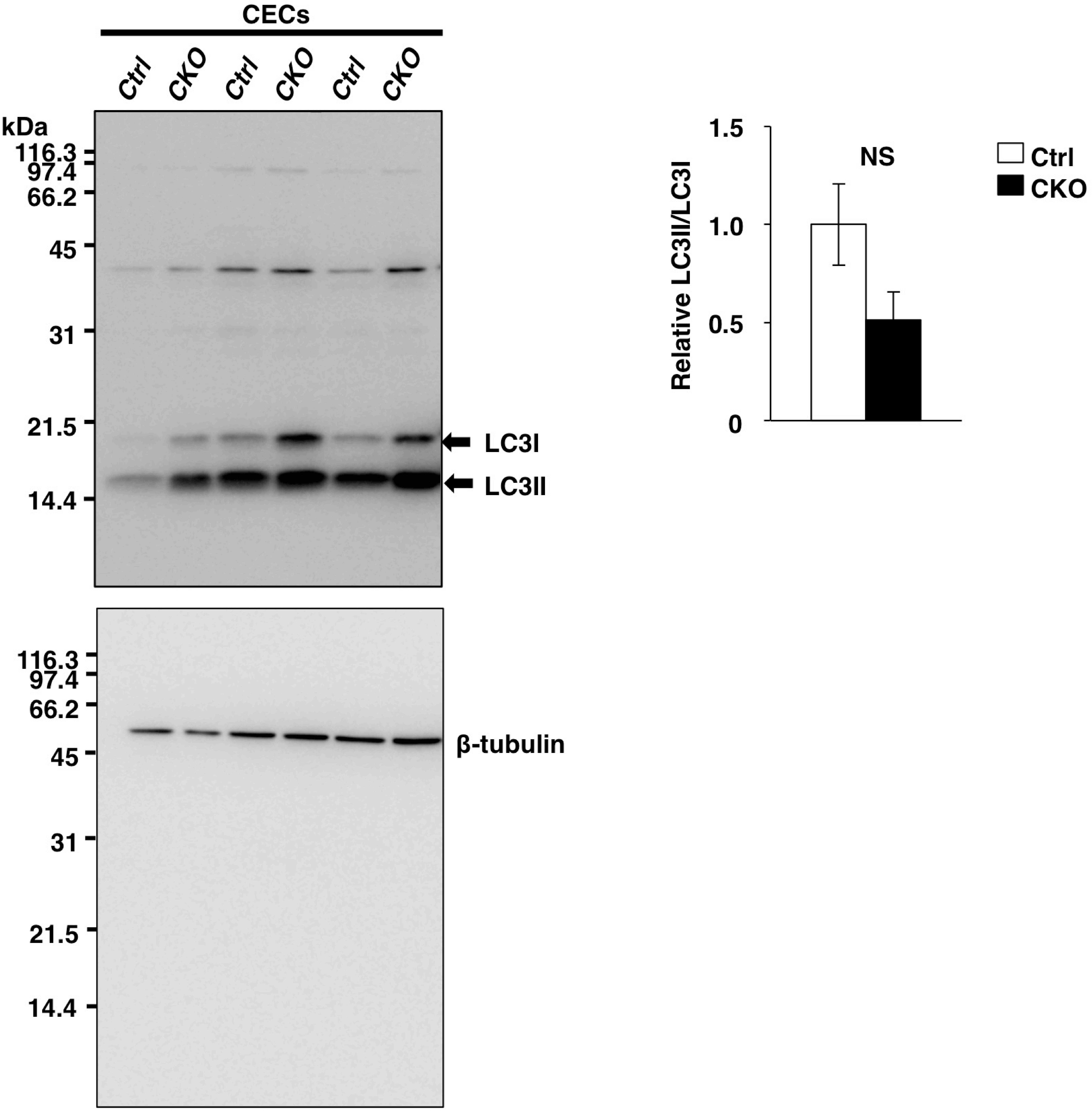

Supplementary Figure S6. Evaluation of autophagy in CECs of Tsc2 CKO mice. Lysates of CECs from control or Tsc2 CKO mice at 10- to 12-week-old were subjected to immunoblot analysis with antibodies to LC3 and to β-tubulin. Representative blots and densitometric analysis of the LC3II/LC3I band intensity ratio are shown, with the quantitative data being expressed relative to the corresponding value for control mice and presented as means ± s.e. from three separate experiments. NS, not significant (Student's *t* test).

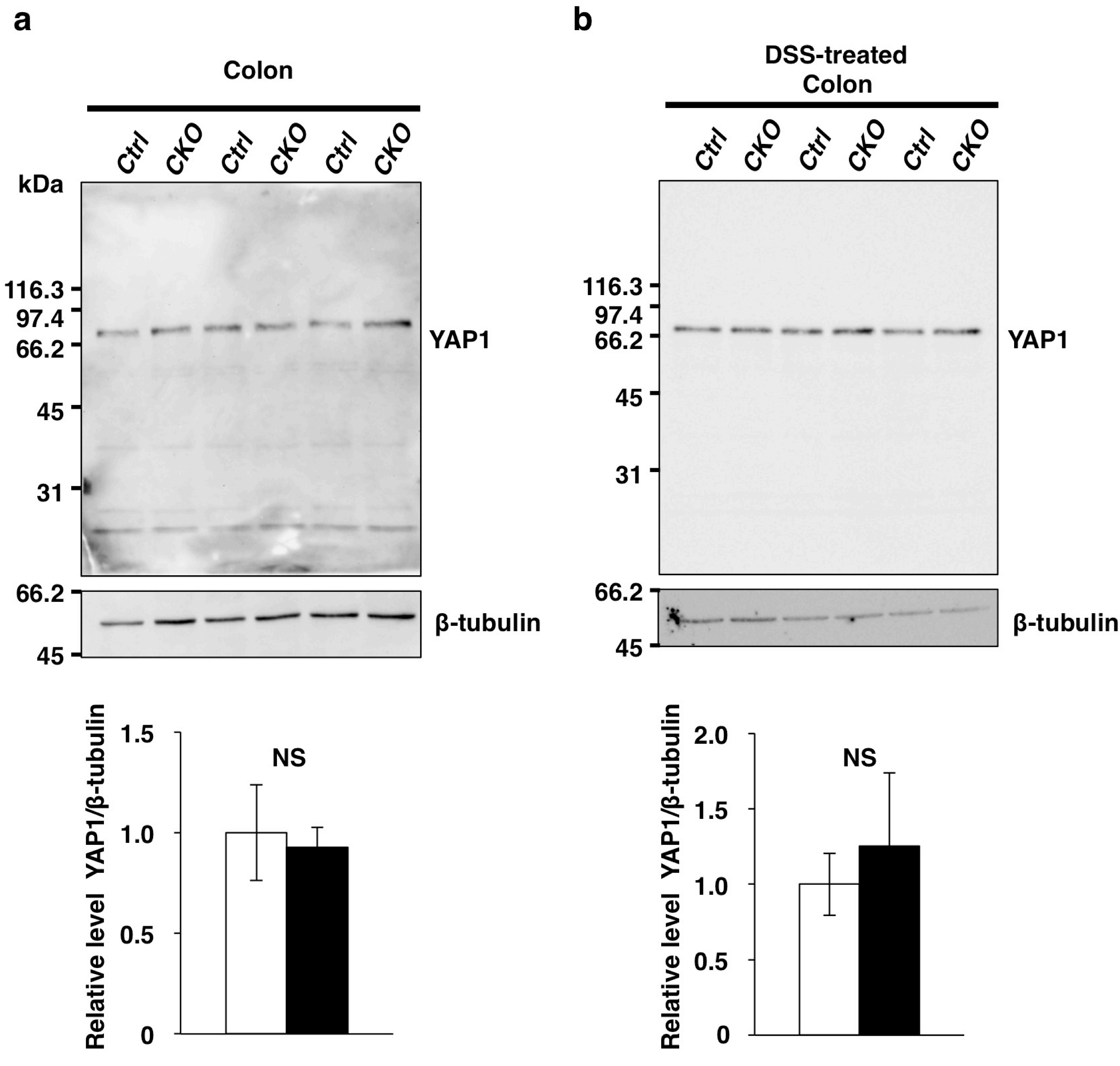

Supplementary Figure S7. Expression of YAP1 in the colon of control and Tsc2 CKO mice before or after DSS-treatment. (**a**) Lysates of the colon from control and Tsc2 CKO mice at 13- to 14-week-old were subjected to immunoblot analysis with antibodies to YAP1 and to β-tubulin. Representative blots as well as densitometric analysis of the YAP1/β-tubulin band intensity ratio are shown, with the quantitative data being expressed relative to the corresponding value for control mice and presented as means ± s.e. from three separate experiments. NS, not significant (Student's *t* test). (**b**) Lysates of the colon from control and Tsc2 CKO mice at 3 days after DSS treatment were subjected to immunoblot analysis with antibodies to YAP1 and to β-tubulin. Representative blots as well as densitometric analysis of the YAP1/β-tubulin band intensity ratio are shown, with the quantitative data being expressed relative to the corresponding value for control mice, and presented as means ± s.e. from three separate experiments. NS, not significant (Student's *t* test).

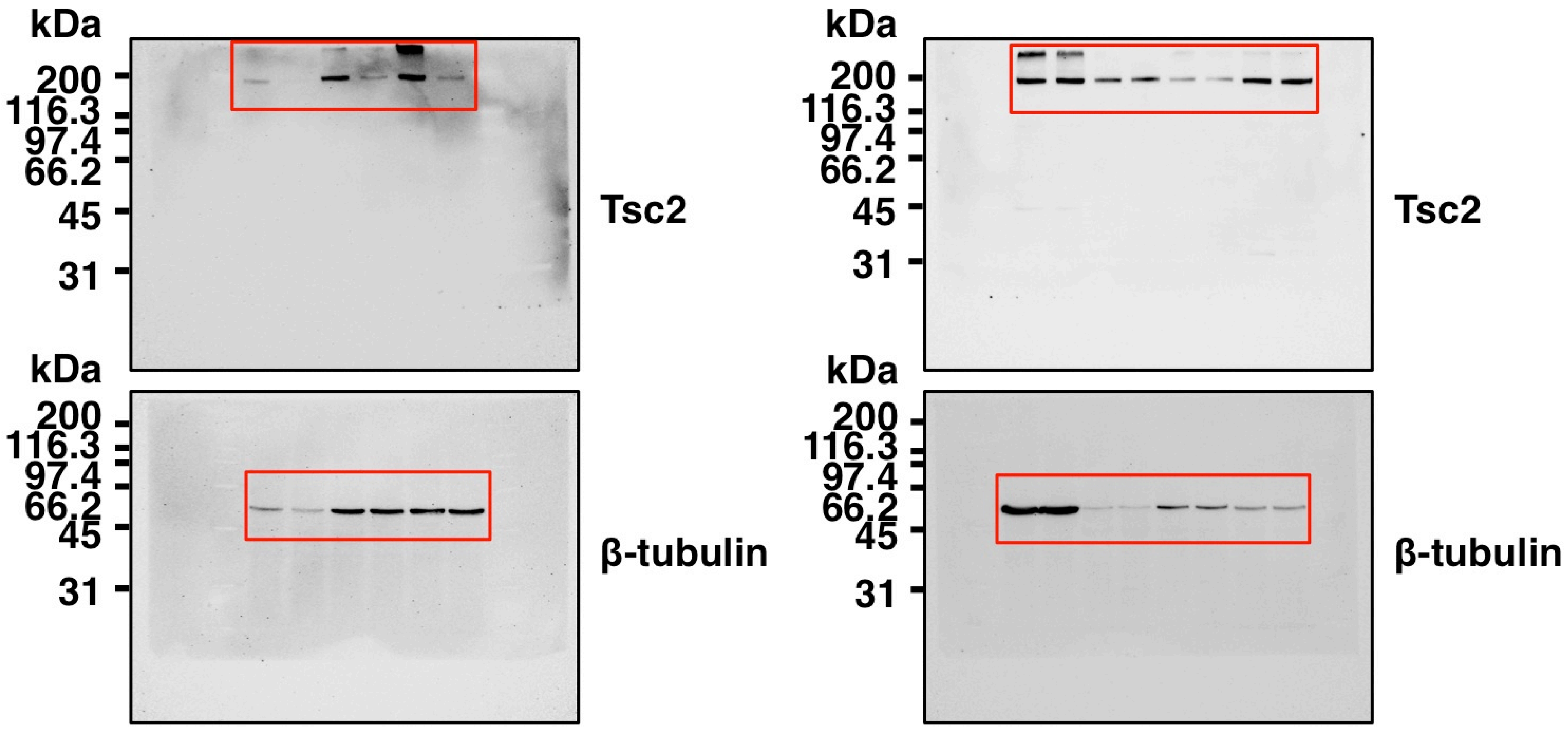

Immunoblot data for Figure 1a

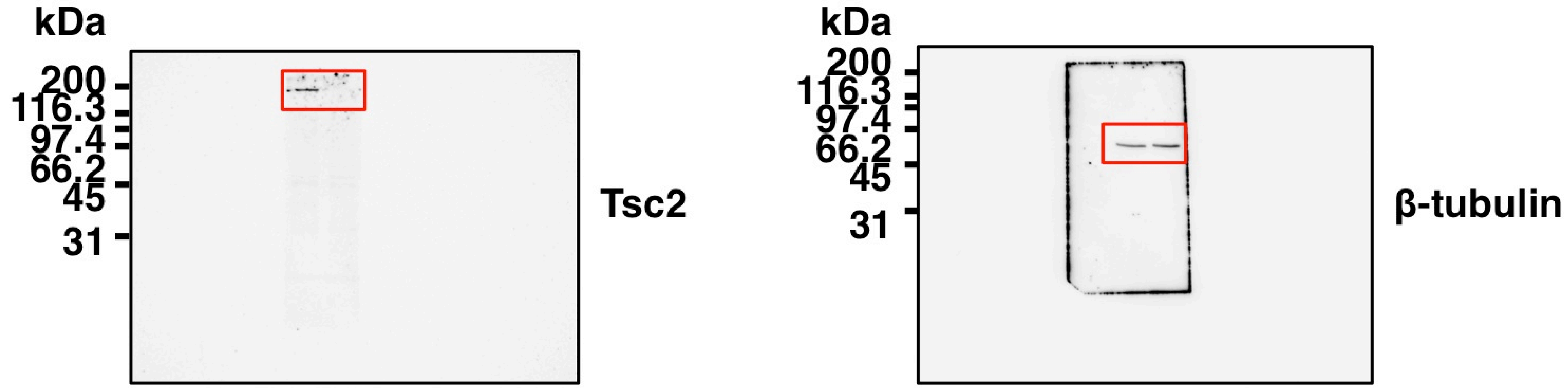

Immunoblot data for Figure 1b

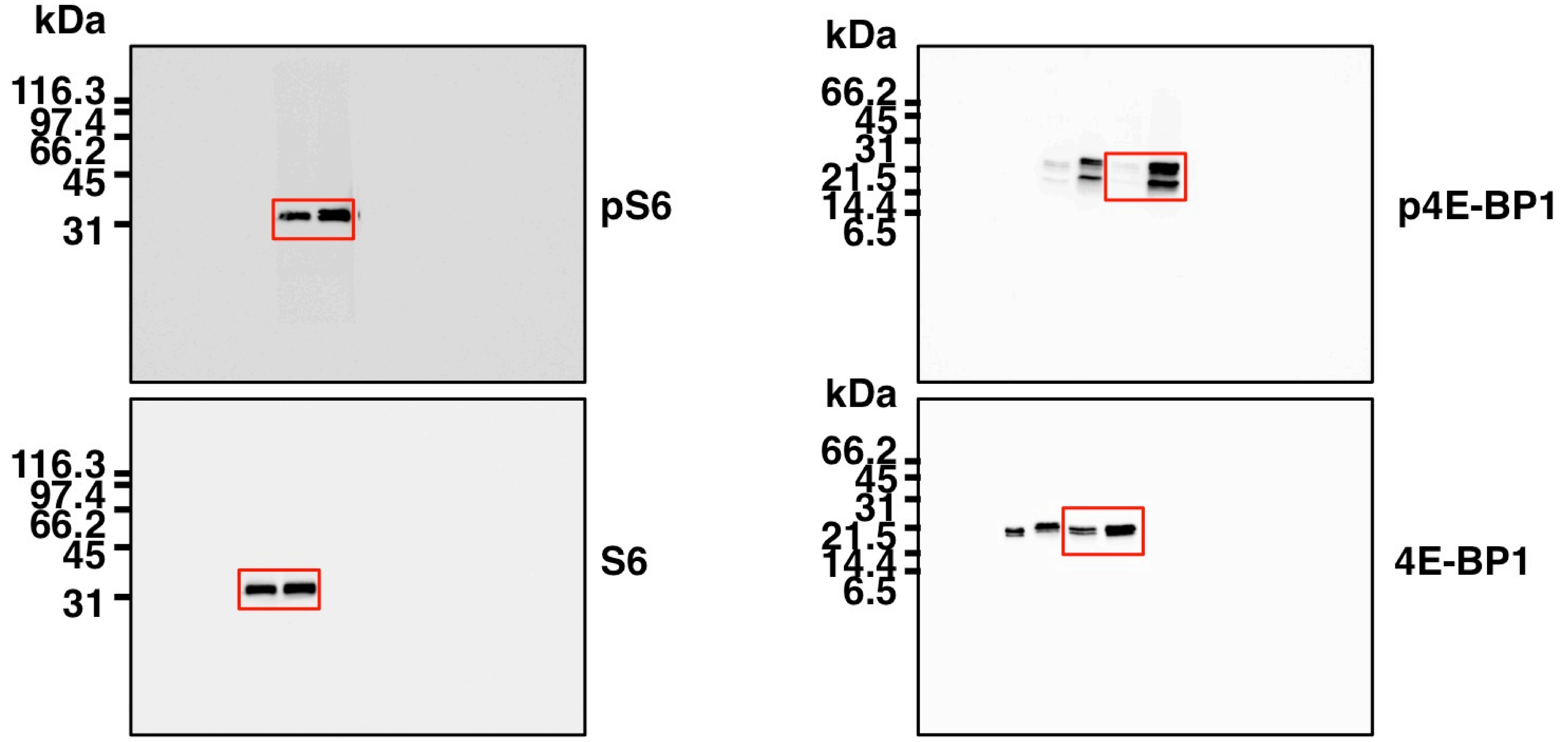

Immunoblot data for Figure 1c

Supplementary Figure S8. Uncropped immunoblot data for Figure 1

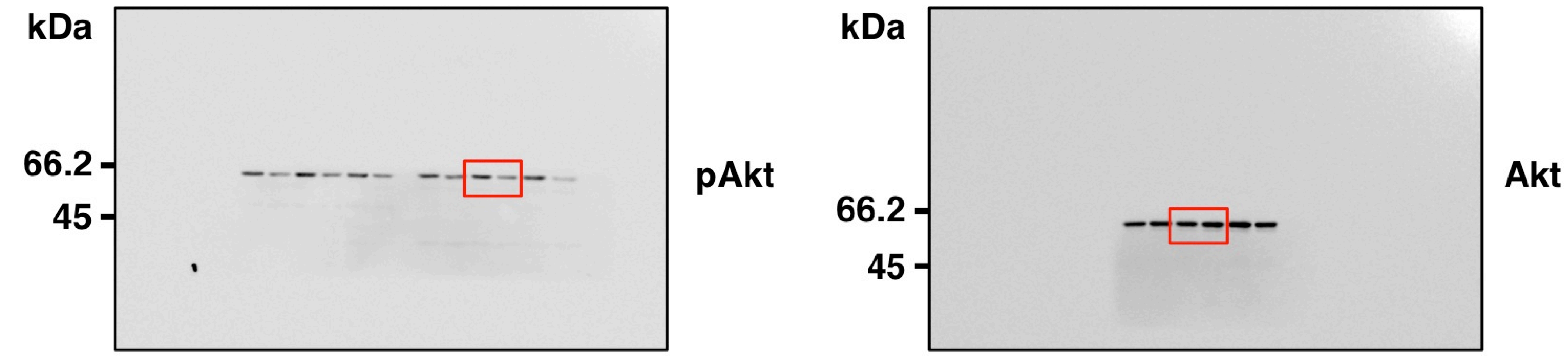

Immunoblot data for Figure 3a

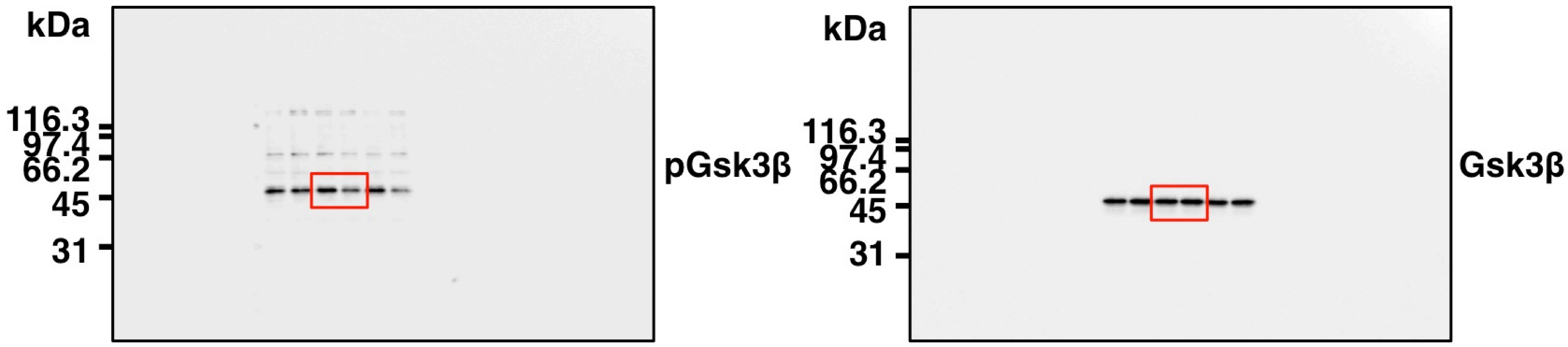

Immunoblot data for Figure 3b

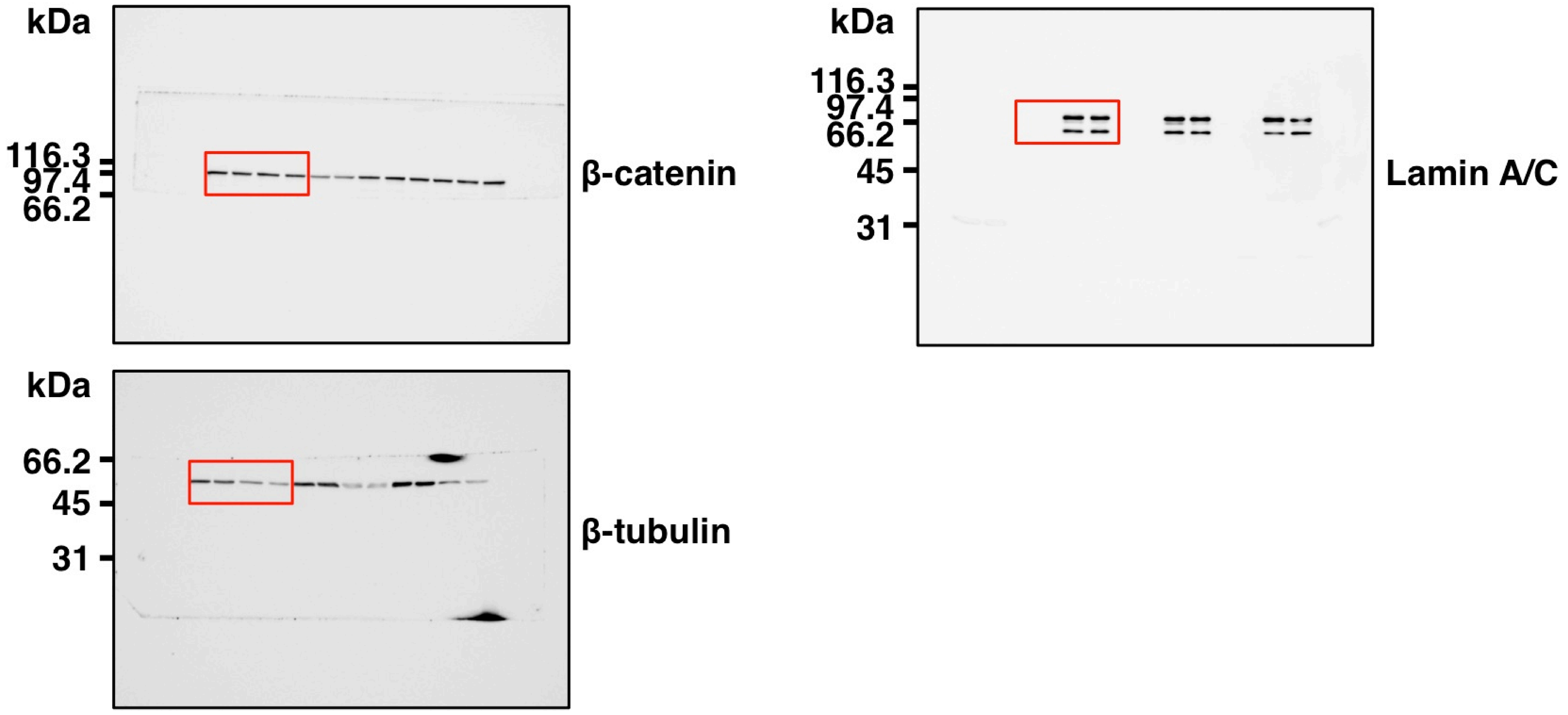

Immunoblot data for Figure 3c

Supplementary Figure S9. Uncropped immunoblot data for Figure 3
